# Identification of compounds that bind the centriolar protein SAS-6 and inhibit its oligomerization

**DOI:** 10.1074/jbc.RA120.014780

**Published:** 2021-01-13

**Authors:** Julia M.C. Busch, Minos-Timotheos Matsoukas, Maria Musgaard, Georgios A. Spyroulias, Philip C. Biggin, Ioannis Vakonakis

**Affiliations:** 1 Department of Biochemistry, University of Oxford, Oxford, United Kingdom; 2 Department of Pharmacy, University of Patras, Patras, Greece

**Keywords:** centrosome, protein-drug interaction, inhibitor, ligand design, biophysics, nuclear magnetic resonance (NMR), protein-protein interaction

## Abstract

Centrioles are key eukaryotic organelles that are responsible for the formation of cilia and flagella, and for organizing the microtubule network and the mitotic spindle in animals. Centriole assembly requires oligomerization of the essential protein spindle assembly abnormal 6 (SAS-6), which forms a structural scaffold templating the organization of further organelle components. A dimerization interaction between SAS-6 N-terminal “head” domains was previously shown to be essential for protein oligomerization *in vitro* and for function in centriole assembly. Here, we developed a pharmacophore model allowing us to assemble a library of low-molecular-weight ligands predicted to bind the SAS-6 head domain and inhibit protein oligomerization. We demonstrate using NMR spectroscopy that a ligand from this family binds at the head domain dimerization site of algae, nematode, and human SAS-6 variants, but also that another ligand specifically recognizes human SAS-6. Atomistic molecular dynamics simulations starting from SAS-6 head domain crystallographic structures, including that of the human head domain which we now resolve, suggest that ligand specificity derives from favorable Van der Waals interactions with a hydrophobic cavity at the dimerization site.

Centrioles are organelles that are widespread in eukaryotes (1, 2, 3) and coordinate a broad spectrum of cellular activities. In animal cells, a pair of centrioles close to the nucleus compose the structured core of the centrosome, which acts as the major microtubule-nucleating center of the cell (4, 5). Centrosomes nucleate microtubules and direct formation of their network, which is essential for intracellular transport processes and organelle positioning relative to the nucleus, and organize the mitotic spindle during cell division, thereby assisting the correct segregation of genetic material. Moreover, centrioles in nonproliferating animal cells and in unicellular eukaryotes dock to the cell membrane, where they act as templating platforms for cilia and flagella involved in motility and sensing (6). The number of centrioles in cells is maintained constant through new organelle assembly at each cell cycle (7, 8), which starts with recruitment of the spindle assembly abnormal protein 6 (SAS-6) to a site adjacent to each preexisting centriole.

SAS-6 is an essential protein for centriole formation (9, 10, 11, 12, 13) and a key component of a scaffold-like structure known as the “cartwheel” (14) or “central tube” (15). The cartwheel forms early in the centriole assembly process and subsequently organizes downstream organelle components (7, 8). Cartwheel formation by SAS-6 depends on large-scale oligomerization of this protein. Crystallographic, EM, atomic force microscopy, and biophysical studies of SAS-6 variants from multiple species have shown that SAS-6 oligomerization is driven by two dimerization interfaces mediated by the protein “head” domain and a homodimeric coiled-coil domain (16, 17, 18, 19, 20, 21, 22, 23). These dimerization interfaces cooperate to form closed rings of SAS-6 proteins in most species or open-ended spiral arrangements in nematodes, both of which have been reconstituted *in vitro* (16, 17, 18, 19, 21, 22, 23). In either case, WT SAS-6 rings and spirals feature 9-fold radial symmetry on average, which matches the radial symmetry of cartwheels and centrioles, whereas engineered SAS-6 variants that form oligomers with altered symmetry lead to the assembly of non9-fold-symmetric organelles (23). Thus, SAS-6 oligomers are thought to impart correct radial symmetry to centrioles, thereby ensuring the formation of functional organelles.

Centriole assembly is a tightly regulated process because the creation of too many or too few organelles can lead to ciliopathies, developmental abnormalities, genomic instability because of unipolar or multipolar mitotic spindles, and potentially cancer (24, 25, 26, 27, 28). The polo-like kinase 4, which initiates centriole duplication by controlling SAS-6 localization to the site of organelle assembly, is considered a promising target for cancer therapy where the underlying causes of the disease involve centrosome overamplification (29). To date, three different small molecule inhibitors selectively target polo-like kinase 4 with nanomolar affinity (30, 31, 32) and block cell proliferation in a number of human cancer lines, including osteosarcoma, cervical carcinoma, and breast, lung, and colon cancer cells (32, 33, 34), likely via centrosome depletion. However, expanding the repertoire of molecular targets for anti-cancer therapeutics is clearly desirable to combat cell desensitization to specific drugs. SAS-6 oligomerization may be such a molecular target, because SAS-6 variants disrupting protein oligomerization prevented centriole assembly in nematodes, algae, insects, and human cells (17, 18, 19, 20).

Here, we evaluate the SAS-6 head domain as a target of small molecules that block domain dimerization and, thus, protein oligomerization. Guided by the SAS-6 head domain structure, we derived a library of chemical fragments that may bind to this domain and tested it against the human, nematode, and algae SAS-6 variants. We discovered one small molecule binding to all three SAS-6 variants, but also another that interacts only with human SAS-6. Combined with crystallographic, NMR, and atomistic molecular dynamics simulations, our work provides a starting point toward developing a specific inhibitor of SAS-6 oligomerization.

## Results

### A virtual screening approach to identify CeSAS-6 head domain interactors

The molecular architecture of SAS-6 proteins is remarkably conserved across species despite large sequence divergence (1), comprising an N-terminal globular head, a 30–50 nm-long coiled coil, and an unstructured C-terminal extension (17, 19). The SAS-6 coiled coil forms a homodimer with the sub-μm dissociation constant (*K_d_*) via an extensive interaction interface and is sufficiently stable to mediate protein dimerization in cells (35). The SAS-6 head domain also self-associates but with lower affinity (*K_d_* of 50–100 μm in vertebrate, nematode, and algae SAS-6) and a smaller dimerization interface compared with the coiled-coil domain (17, 19). Thus, the optimal target for small molecules aimed at disrupting SAS-6 oligomerization may be the weaker and smaller dimerization interface of the SAS-6 head domain.

**Figure 1:**
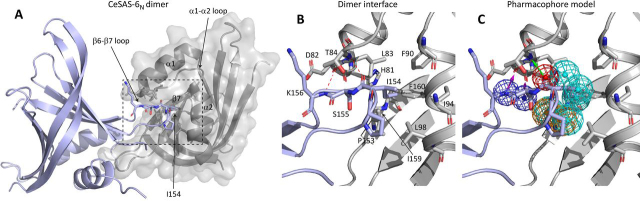
**A pharmacophore model of CeSAS-6_N_ dimerization.***A*, the dimer of CeSAS-6_N_, where the β6-β7 loop of one domain (*light blue*) protrudes into a hydrophobic pocket of the second domain (*gray*) formed by residues on α1, α2, and β7. Derived from PDB ID 3PYI. *B*, magnified view of the dimerization interface, corresponding to the boxed area in (*A*), with key residues annotated and hydrogen bonding interactions show as *dashed red lines*. *C*, a structure-based pharmacophore model depicting the interactions mapped. Hydrophobic features are depicted in *cyan*, aromatic in *orange*, hydrogen bond donors in *blue*, and a hydrogen bond acceptor in *red*. *Arrows* depict the direction of the hydrogen bond vectors (*purple* from donors and *green* from acceptor).

To identify compounds that would disrupt the SAS-6 head domain dimerization, we established a virtual screening process starting from the head domain dimer described for *Caenorhabditis elegans* SAS-6 (CeSAS-6_N_, residues 1–168) (17). Comparison of SAS-6 crystallographic structures suggests that the head domain mode of dimerization is highly conserved (Fig. S1). In the case of CeSAS-6_N_, dimerization involves the domain β6-β7 loop, which is inserted deeply into a hydrophobic cavity of the second monomer (Fig. 1, *A* and *B*). At the tip of β6-β7, a single hydrophobic amino acid (Ile-154) is key for protein association. Ile-154 substitution by charged residues or glycine disrupts CeSAS-6_N_ dimerization and abrogates protein function (17); conversely, Ile-154 substitution by a larger hydrophobic amino acid (I154W) enhances protein association by ∼20-fold (16). Further elements of the dimerization interface are backbone interactions between loop residues Ser-155 and Lys-156 and between residues Asp-82 and Thr-84 in the α1-α2 linker that forms the upper rim of the dimerization hydrophobic pocket. Additionally, Pro-153 likely stabilizes Ile-154 positioning (Fig. 1*B*).

These elements of the dimerization interaction were used to inform a structure-based pharmacophore model in which the CeSAS-6 tetrapeptide Pro-Ile-Ser-Lys (residues 153–156) of the β6-β7 loop was considered as “ligand” and the second protein monomer as “receptor”. The pharmacophore model comprises a set of features, mapped in space, that if satisfied by a small molecule would be suggestive of CeSAS-6_N_ binding. Features mapped included the two hydrophobic contacts of the Ile-154 aliphatic group with the side chains of Phe-90, Ile-94, Leu-98, Ile-159, and Phe-160, which comprise the hydrophobic cavity (Fig. 1*C*). Two hydrogen bond donors were mapped to interact with the carbonyl atoms of His-81 and Asp-82, as well as a hydrogen bond acceptor directed toward the Thr-84 NH atom. The pharmacophore was complemented with one more hydrophobic feature in the cavity, close to Leu-83, and two mixed aromatic ring/hydrophobic features interacting with Leu-98 and Ile-159. We prepared a ligand data set comprising ∼6 million commercially available compounds from the ZINC database, with 250 alternative conformations generated for each compound. All conformers were rigid body–fitted to subgroups of at minimum five features of the pharmacophore model (see “Experimental procedures”) and ranked by goodness of fit. The top-scored ∼2,000 compounds were further filtered according to chemical diversity using the Tanimoto coefficient (36) and by visual inspection to derive a final library of 37 compounds (A1–D1, Table S1) that may associate with the hydrophobic cavity at the CeSAS-6_N_ dimerization interface.

### NMR-based screen confirms small molecule binding to CeSAS-6_N_

We initially assessed the solubility of compounds A1–D1 in aqueous buffer by visual inspection of precipitation following compound transfer from 100% v/v DMSO to 5% v/v DMSO and 2 mm final concentration. Further, we recorded ^1^H NMR spectra of each compound in 5% v/v DMSO. Of the 37 compounds tested, eight were insoluble in aqueous buffer as judged by the absence of resonances in ^1^H NMR spectra, whereas five additional compounds showed decreased solubility as evidenced by precipitation but were still detectable in ^1^H NMR spectra (Table S1).

We then recorded ^1^H-^15^N heteronuclear single quantum coherence (^1^H-^15^N HSQC) NMR spectra of ^15^N-labeled CeSAS-6_N_ in isolation and in the presence of 2 mm nominal concentration of compounds. In such spectra, the position of peaks, corresponding to individual protein amino acids, is sensitive to the amino acid chemical environment and, hence, to binding events. These binding events can be mapped back to the protein structure if the identity of NMR peaks has been assigned, as is the case for CeSAS-6_N_ (37). To lessen the likelihood that compounds targeting the hydrophobic cavity of CeSAS-6_N_ are out-competed by the β6-β7 loop as a result of protein dimerization, we employed a monomeric variant of this protein, CeSAS-6_N_ Δ103–130 I154E. Previous work has shown that the I154E mutation abolishes dimerization (17), whereas deletion of residues 103–130, which form a long unstructured loop, does not affect the domain structure (16) and improves the resolution of NMR spectra (37). Thus, these protein changes leave the hydrophobic pocket of the dimerization interface unaltered and solvent-exposed.

**Figure 2:**
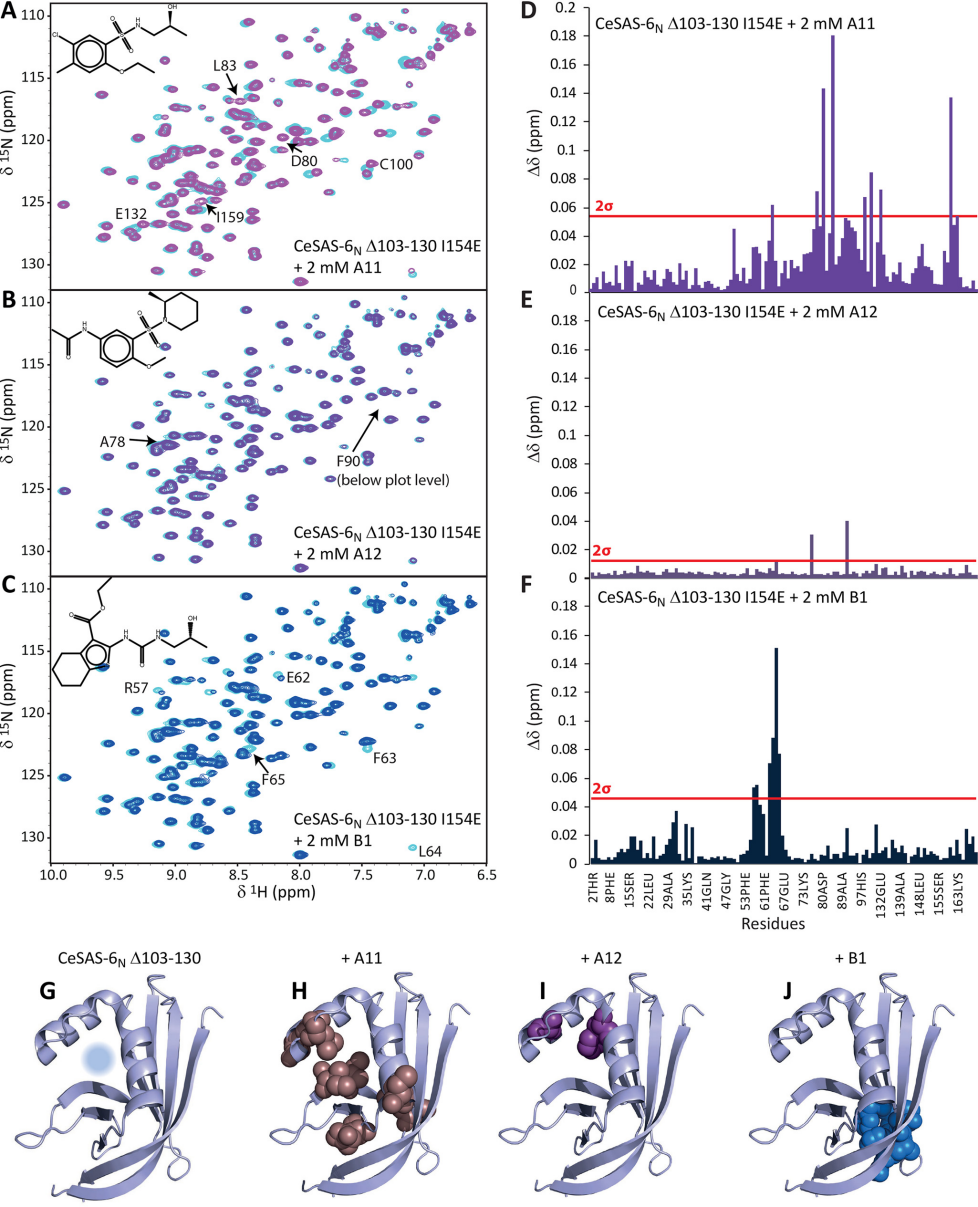
**NMR assay of CeSAS-6_N_ small molecule interactors.***A*–*C*, overlay of NMR ^1^H-^15^N HSQC spectra produced by CeSAS-6_N_ Δ103–130 I154E alone (*cyan*) or in the presence of 2 mm A11 (*A*), A12 (*B*), or B1 (*C*) compounds. The chemical structures of the compounds are inlaid, and CeSAS-6_N_ resonances exhibiting the most significant changes upon addition of compounds are labeled. *D*–*F*, per-residue quantification of combined changes in ^1^H and ^15^N chemical shifts of CeSAS-6_N_ Δ103–130 I154E resonances upon addition of 2 mm A11 (*D*), A12 (*E*), or B1 (*F*) compounds. A measure of two standard deviations of all changes observed is shown as a *red line*, indicating amino acids that experienced the strongest perturbations upon compound addition. *G*, CeSAS-6_N_ Δ103–130 monomer structure with the dimerization site targeted by compounds indicated by a *light blue circle*, derived from PDB ID 4G79. *H*–*J*, amino acid residues strongly perturbed by addition of compounds A11 (*H*), A12 (*I*), or B1 (*J*), shown in sphere representation. Only A11 produces changes in amino acids surrounding the targeted site, suggesting compound binding at the hydrophobic cavity of the dimerization interface.

Comparison of ^1^H-^15^N HSQC NMR spectra from CeSAS-6_N_ Δ103–130 I154E alone and with compounds allowed an initial qualitative differentiation of compound affinity. Fig. 2, *A*–*F* show exemplar NMR spectra obtained upon addition of compounds A11, A12, and B1 to CeSAS-6_N_ Δ103–130 I154E and the per-residue quantification of spectral changes. As seen there, addition of compounds A11 and B1 to CeSAS-6_N_ Δ103–130 I154E caused notable changes in NMR peak positions, indicative of potential binding, whereas addition of A12 did not have a visible effect on NMR spectra. Mapping the most strongly perturbed amino acids to the CeSAS-6_N_ structure reveals that A11 binding affects residues at the vicinity of the hydrophobic cavity at the dimerization interface (Fig. 2, *G* and *H*), that A12 causes few observable changes to the protein (Fig. 2*I*), and that residues affected by B1 binding are not in the target pocket (Fig. 2*J*). Overall, among the 29 soluble or partly soluble compounds tested, A11 was the only compound that perturbed NMR spectra in a pattern indicating binding to the CeSAS-6_N_ target pocket (Fig. S2).

We attempted to estimate the binding affinity of A11 to CeSAS-6_N_ by recording a series of ^1^H-^15^N HSQC NMR spectra and assessing the extent of peak changes as a function of A11 concentration. As shown in Fig. S3, NMR spectral perturbations continued to increase in A11 titrations and did not reach saturation even at 2 mm compound concentration, suggesting a binding affinity in the millimolar range. Furthermore, we examined whether A11 binding altered the dynamic behavior of CeSAS-6_N_. We recorded heteronuclear {^1^H}-^15^N NOE ratios, which are sensitive to picosecond-nanosecond timescale motions (38), in the presence of A11. As shown in Fig. S4, addition of A11 did not have a significant impact on overall protein flexibility, which may be because the interaction of A11 with CeSAS-6_N_ is very weak (Fig. S3) and, thus, rather transient.

### Compound A11 disrupts CeSAS-6 oligomerization

**Figure 3:**
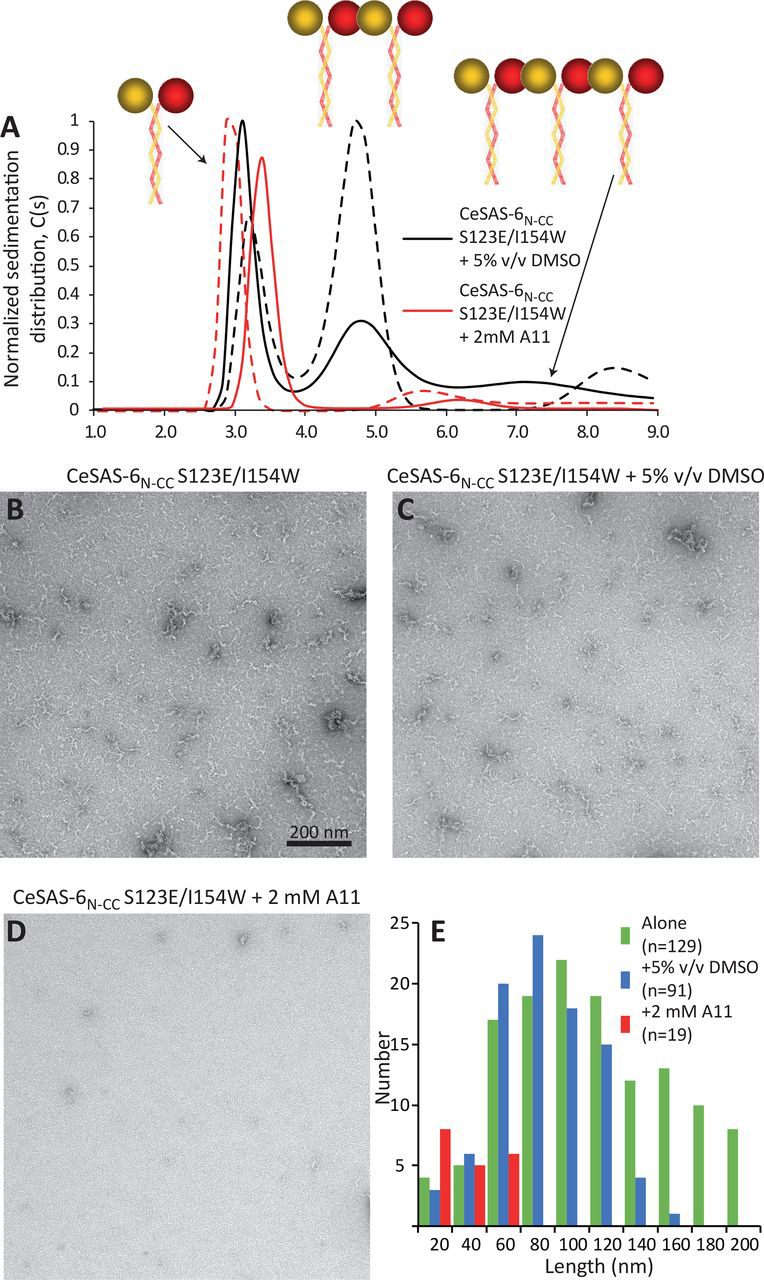
**Assays of CeSAS-6_N-CC_ oligomerization.***A*, sedimentation velocity analysis of CeSAS-6_N-CC_ S123E/I154W in the presence of 5% v/v DMSO (*black solid and dashed lines*) or upon addition of 2 mm A11 (*red solid and dashed lines*). *Solid versus dashed lines* correspond to two independent repeats of the assay. The molecular architecture of CeSAS-6_N-CC_ and the oligomeric state of different sedimentation species are shown schematically on top (*spheres*: head domains; *zigzag lines*: coiled-coil segments). Stable CeSAS-6 dimers are mediated by the coiled-coil segment. Addition of A11 reduces the prevalence of higher oligomeric species, which necessitate interactions between head domains. *B*–*D*, representative electron micrographs of CeSAS-6_N-CC_ S123E/I154W spiral assemblies formed at 1 mg/ml protein concentration and standard buffer (*B*) or in the presence of 5% v/v DMSO (*C*) or 2 mm A11 (*D*). *Common scale bar* in *panel B* 200 nm. *E*, plot of assembly length *versus* number of occurrences from the CeSAS-6_N-CC_ S123E/I154W samples shown in (*B*–*D*). Addition of A11 strongly reduces the number and length of visible assemblies.

A11 is the first small molecule shown to bind the CeSAS-6_N_ hydrophobic cavity at the dimerization interface. To assess whether such binding disrupts protein oligomerization, we performed sedimentation velocity analytical ultracentrifugation (AUC) experiments monitored by optical interference. For these experiments, a CeSAS-6 construct that includes both the N-terminal domain and a section of the coiled-coil domain (CeSAS-6_N-CC_; S123E/I154W, residues 1–215) was used, which can form oligomers if both dimerization interfaces engage in self-association interactions (Fig. 3*A*). Furthermore, the construct bore two mutations that stabilized the protein in solution (S123E) (17) and enhanced the head domain dimerization affinity (I154W) (16), thereby leading to stronger oligomers. The latter was deemed essential because addition of even small amounts of DMSO, in which compounds were dissolved, had a negative effect on higher-order protein oligomerization (Fig. S5A). We observed that addition of 2 mm A11 in AUC assays reduced the prevalence of large protein oligomers such as tetramers derived from two CeSAS-6_N-CC_ coiled-coil dimers interacting via their head domains (Fig. 3*A*). In contrast, addition of 2 mm A12, which did not show CeSAS-6_N_ binding in NMR assays, had no significant effect on CeSAS-6_N-CC_ oligomerization (Fig. S5B).

To further assess the effect of A11 on CeSAS-6 oligomerization, we imaged the formation of open-ended spiral assemblies by this protein using EM. We previously showed that such assemblies form readily on carbon-coated grids, likely assisted by protein adsorption on the grids (16). As shown in Fig. 3, *B* and *E*, samples of CeSAS-6_N-CC_ S123E/I154W alone produced multiple long-spiral assembles, whereas addition of 5% v/v DMSO slightly reduced both the number and length of these assemblies (Fig. 3, *C* and *E*). However, addition of 2 mm A11 in these assays strongly suppressed formation of spirals, resulting in far fewer and shorter assemblies (Fig. 3, *D* and *E*). We conclude that A11 prevents CeSAS-6 oligomerization likely by competing with the CeSAS-6_N_ β6-β7 loop for binding to the hydrophobic pocket of the head domain dimerization interface.

### Binding of library compounds to SAS-6 orthologues

The pharmacophore features used to select compounds for binding to the CeSAS-6_N_ dimerization interface, which included hydrogen bonds directed against protein backbone atoms and the hydrophobic nature of the interface, are common across SAS-6 orthologues. We thus posited that compounds selected using the pharmacophore model of CeSAS-6_N_ may also bind head domains from other variants of this protein. To test this hypothesis, we recorded ^1^H-^15^N HSQC NMR spectra of *Chlamydomonas reinhardtii* and human SAS-6 head domains (CrSAS-6_N_, residues 1–159 and HsSAS-6_N_, residues 1–152, respectively) in the presence of 2 mm of library compounds. Similar to earlier assays of CeSAS-6_N_, we employed dimerization-impaired variants of these domains (*C. reinhardtii* SAS-6_N_ (CrSAS-6_N_) F145E and HsSAS-6_N_ F131E) (17) to ensure that the hydrophobic cavity of the dimerization interface is solvent-exposed.

**Figure 4:**
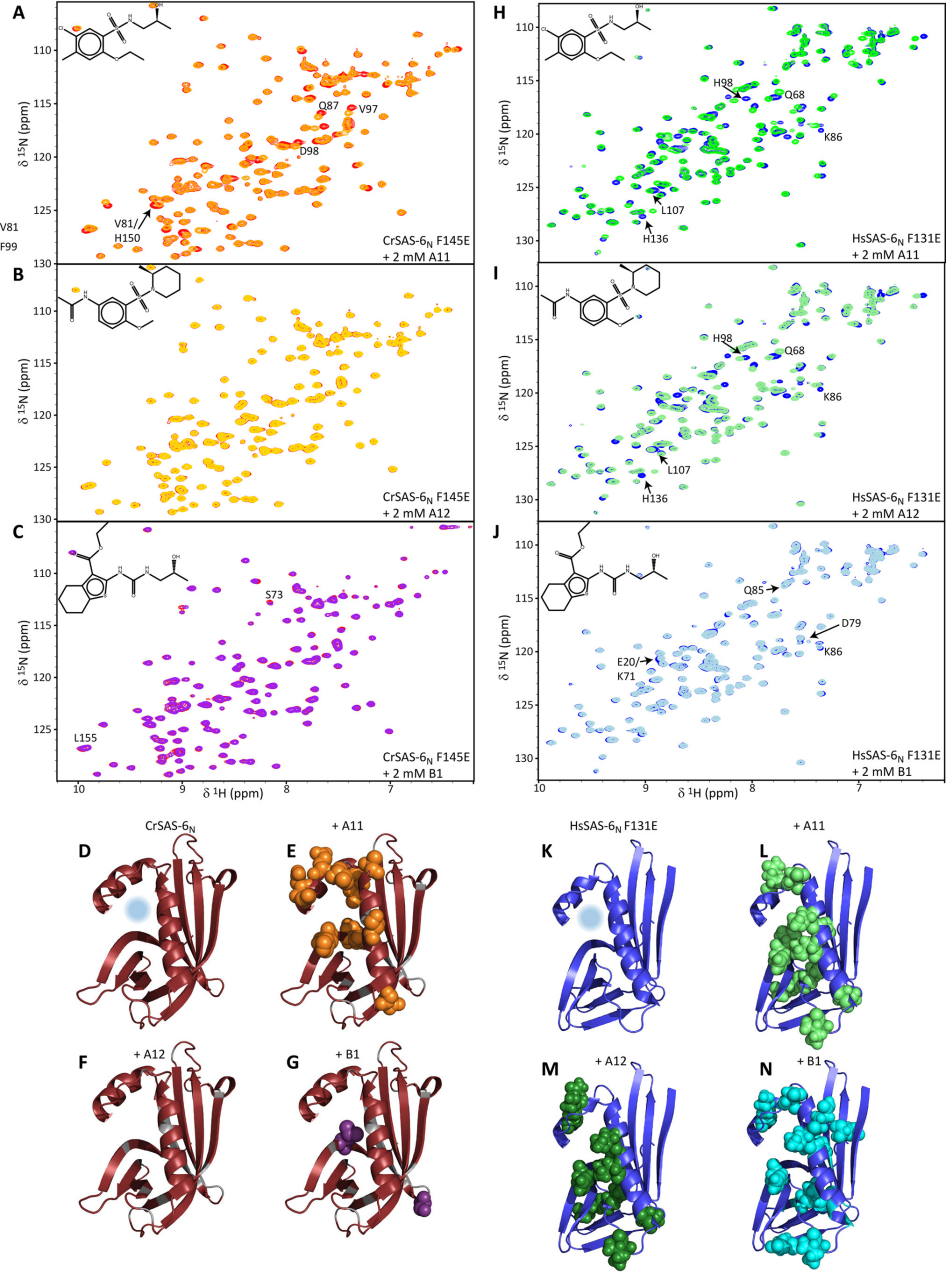
**NMR assays of CrSAS-6_N_ and HsSAS-6_N_ compound binding.***A*–*C*, overlay of NMR ^1^H-^15^N HSQC spectra produced by CrSAS-6_N_ F145E alone (*red*) or in the presence of 2 mm A11 (*A*), A12 (*B*), or B1 (*C*) compounds. The chemical structure of compounds is inlaid, and CrSAS-6_N_ resonances exhibiting the most significant changes upon addition of compounds are labeled. *D*, CrSAS-6_N_ monomer structure with the dimerization site indicated by a *light blue circle*, derived from PDB ID 3Q0Y. *E*–*G*, amino acid residues strongly perturbed by addition of compounds A11 (*E*), A12 (*F*), or B1 (*G*), shown in sphere representation. Only A11 produces changes in amino acids surrounding the targeted site, suggesting compound binding at the hydrophobic cavity of the dimerization interface. *H*–*N*, similar NMR spectra of HsSAS-6_N_ F131E (*H*–*J*, *blue*) with compounds A11, A12, and B1, and structure representations of the HsSAS-6_N_ dimerization site (*K*) and residues affected by compound binding (*L*–*N*). The HsSAS-6_N_ structure was resolved as part of this study (Fig. S8 and Table S2). In contrast to CeSAS-6_N_ and CrSAS-6_N_, both A11 and A12 bind the dimerization site of HsSAS-6_N_.

Analysis of CrSAS-6_N_ F145E NMR spectra showed that, similar to CeSAS-6_N_, A11 yielded the strongest perturbations of amino acid resonances, suggesting binding of this compound to the head domain (Fig. 4, *A*–*C*). We assigned the NMR spectra of CrSAS-6_N_, allowing us to quantitate these changes on a per-residue basis (Fig. S6, A–C and Fig. S7A) and locate the amino acids affected by A11 binding on the domain structure. As shown in Fig. 4, *D* and *E*, addition of A11 perturbed amino acids surrounding the hydrophobic cavity of the CrSAS-6_N_ dimerization site, whereas addition of other compounds, such as A12 or B1, produced only small changes in this domain (Fig. 4, *F* and *G*). Similar analysis of HsSAS-6_N_ F131E NMR spectra revealed that A11 also bound the human orthologue of the SAS-6 head domain (Fig. 4*H*). However, in contrast to previous titrations in CeSAS-6_N_ and CrSAS-6_N_, we observed that A12 bound to HsSAS-6_N_ and yielded perturbations in the NMR spectra comparable with those of A11 (Fig. 4*I*). Other compounds in the library, such as B1 (Fig. 4*J*), did not produce analogous changes on HsSAS-6_N_ F131E spectra.

To elucidate the location of A11 and A12 binding to HsSAS-6_N_, we assigned the NMR spectra of this domain and determined the crystallographic structure of HsSAS-6_N_ F131E at 1.46 Å resolution. Comparison of the HsSAS-6_N_ head domain structure to that of other resolved SAS-6 variants revealed that the human protein is most similar to CrSAS-6_N_ and the *Danio rerio* SAS-6 head domain, with sub-0.7 Å root mean square deviation (RMSD) of C_α_ atom positions (Fig. S8). In contrast, the HsSAS-6_N_ structure diverged more strongly from that of CeSAS-6_N_ (∼2.8 Å C_α_ RMSD), largely driven by repositioning of the α1 and α2 helices. We assigned the HsSAS-6_N_ F131E NMR spectra and quantified the spectral changes upon addition of compounds on a per-residue basis (Fig. S6, D–F and Fig. S7B), which allowed us to locate the most affected amino acids on the domain structure. As shown in Fig. 4, *K*–*M*, both A11 and A12 perturb amino acids surrounding the dimerization interface, whereas addition of other compounds did not produce a similar pattern (Fig. 4*N*). We conclude that A11 binds at the hydrophobic cavity of the SAS-6 head domain dimerization interface in a promiscuous manner but that, interestingly, A12 displays species selectivity binding only at the relevant cavity of the human orthologue.

### Molecular dynamics simulations suggest binding determinants of A11 and A12 to SAS-6 head domains

**Figure 5:**
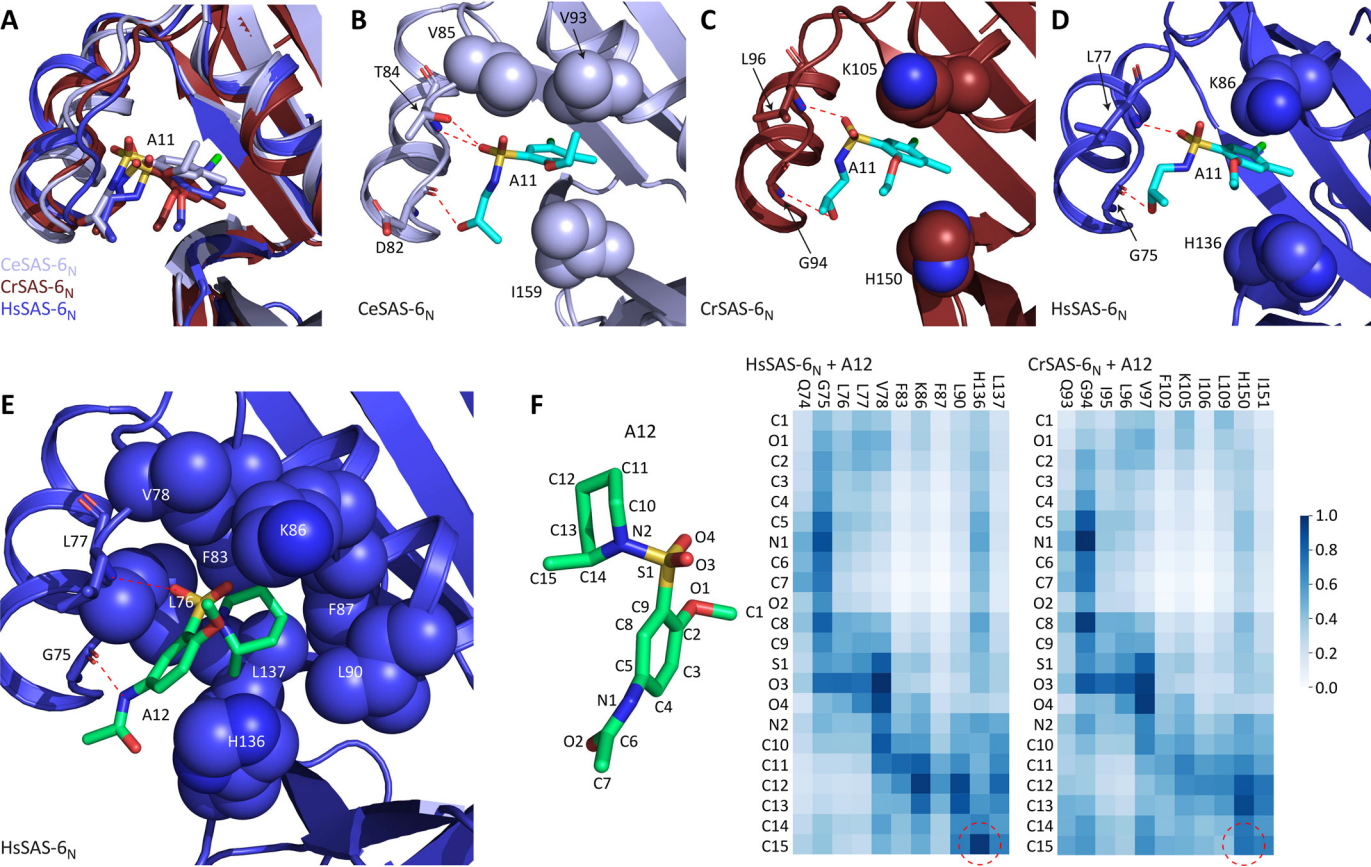
**MD simulations of A11 and A12 binding to SAS-6 head domains.***A*, superposition of representative states from MD simulations of CeSAS-6_N_ (*light blue*), CrSAS-6_N_ (*red*), and HsSAS-6_N_ (*blue*) with A11. The ligand, shown as *sticks* and with carbon atoms colored similar to the respective protein, adopts a consistent binding pose relative to head domains (schematic representation), with the compound aromatic group buried in the hydrophobic cavity of the head domain dimerization site. *B*, key interactions forming between A11 and CeSAS-6_N_ seen in MD simulations. Hydrogen bonds are shown as *red dashed lines*. A11 and protein amino acids participating in hydrogen bonds are shown in *stick* representation. The sidechains of amino acids involved in hydrophobic interactions are shown as *spheres*. The ligand is shown as *sticks* with carbon atoms in *cyan*. *C*–*E*, similar views of interactions between A11 and CrSAS-6_N_ (*C*) or HsSAS-6_N_*(D*), and A12 with HsSAS-6_N_ (*E*). The A12 ligand carbon atoms are colored *light green*. *F*, ligand contact maps from the HsSAS-6_N_ and CrSAS-6_N_ MD simulations with A12. A12 atom nomenclature is shown alongside a *stick* representation of the ligand (*right panel*). The HsSAS-6_N_ (*middle panel*) and CrSAS-6_N_ (*left panel*) maps show close contacts between protein residues and ligand atoms as annotated. Different densities (*blue hues*) denote the occurrence of close contacts during the simulation, normalized to the most frequently observed contact (assigned value of 1, *dark blue*). *Dashed circles* highlight close contacts of the A12 methyl group in the HsSAS-6_N_ simulation that are absent in CrSAS-6_N_.

To understand the binding of compounds A11 and A12 to SAS-6 head domains, and the origins of A12 species selectivity, we performed atomistic molecular dynamics (MD) simulations. A11 and A12 were docked in the hydrophobic cavity of the CeSAS-6_N_ dimerization site in an initial binding pose consistent with the pharmacophore model, and to those of CrSAS-6_N_ and HsSAS-6_N_ using docking approaches. We performed four replicate MD simulations, each 0.5 μs long, in an aqueous environment. A11 simulations revealed a broadly stable and consistent binding pose of the compound in the dimerization cavity (Fig. 5*A*, Fig. S9, A–C, and Movies S1–S3), although excursions from this position were observed in HsSAS-6_N_ (Fig. S9B and Movie S2). The most common A11 binding pose involved the compound aromatic group inserted deep in the hydrophobic cavity led by the chloride atom and forming close interactions with the sidechains of Val-85, Val-93, and Ile-159 in CeSAS-6_N_ (Fig. 5*B*). Similar hydrophobic interactions were observed in MD simulations of A11 with CrSAS-6_N_ and HsSAS-6_N_ (Fig. 5, *C* and *D*). Further, the simulations suggested the presence of stable hydrogen bonds forming between A11 and the α1-α2 loop of SAS-6 head domains. Specifically, the A11 sulfonyl oxygens participated in two hydrogen bonding interactions with the backbone amide and hydroxyl hydrogens of CeSAS-6_N_ Thr-84, and the A11 hydroxyl group formed a third hydrogen bond with the backbone carbonyl of Asp-82 (Fig. 5*B*). Although the Thr-84 equivalent residue in CrSAS-6_N_ and HsSAS-6_N_ is a leucine (Leu-96 and Leu-77, respectively), two hydrogen bonding interactions between an A11 sulfonyl oxygen and the backbone amide of this leucine, and between the A11 hydroxyl group and the backbone carbonyl of Gly-75 (HsSAS-6_N_) or Gly-94 (CrSAS-6_N_), were sufficient to maintain a stable binding pose (Fig. 5, *C* and *D*). This suggests that the CeSAS-6_N_ Thr-84 side-chain–mediated hydrogen bond is not critical for A11 binding. Excursions from this binding pose in the MD simulations of A11 with HsSAS-6_N_ maintained the interactions of the compound aromatic group with the hydrophobic cavity at the dimerization site but involved the formation of new hydrogen bonding interactions with residues of helix α2 (Movie S2).

Compared with A11, no stable binding pose was observed in MD simulations of A12 with CeSAS-6_N_, as evidenced by high and fluctuating ligand RMSD values (Fig. S9D and Movie S4), which is consistent with the lack of A12 binding to this SAS-6 head domain in NMR assays (Fig. 2, *B* and *D*). In contrast, A12 adopted a semi-stable binding pose to HsSAS-6_N_ (Fig. S9E and Movie S5), in agreement with binding observed by NMR (Fig. 4*I* and Fig. S6E). A12 binding involved fluctuating interactions of the methylpiperidine moiety with multiple residues of the HsSAS-6_N_ dimerization cavity (Leu-76, Val-78, Phe-83, Lys-86, Phe-87, Leu-90, His-136, and Leu-137; Fig. 5*E*), compared with A11 where similar but tighter interactions were formed by the compound aromatic group (Fig. 5*D*). We postulate that the methoxy and acetamide decorations of the A12 aromatic group prevented its stable insertion in the HsSAS-6_N_ hydrophobic cavity. The A12 methylpiperidine moiety was somewhat mobile in the hydrophobic cavity as a result of rotations around the sulfonyl-methylpiperidine bond (Fig. S9D and Movie S5), albeit with a preference on methyl group orientation (see below). We observed the formation of two stable hydrogen bonds between the A12 sulfonyl group oxygen and the HsSAS-6_N_ Leu-77 amide and between the A12 acetamide and the Gly-75 carbonyl atoms (Fig. 5*E*).

Although dimerization cavities of SAS-6 head domains are consistently hydrophobic, we noted that only three of eight HsSAS-6_N_ amino acids involved in interactions with the A12 methylpiperidine moiety are strictly conserved in CeSAS-6_N_ (Fig. S8G). These changes in amino acid sidechains may account for the lack of favorable methylpiperidine packing in the dimerization cavity of CeSAS-6_N_, leading to high compound mobility in simulations. Consistent with this analysis, MD simulations of A12 in complex to CrSAS-6_N_, where five of eight methylpiperidine-binding amino acids are conserved, showed relatively stable compound binding (Fig. S9F and Movie S6). However, in these CrSAS-6_N_ simulations the A12 methylpiperidine moiety showed no clear positioning preference, whereas in HsSAS-6_N_ simulations the moiety methyl group was primarily directed toward Leu-76, His-136, and Leu-137 (Fig. 5*F*). Two of these three HsSAS-6_N_ amino acids are substituted in CrSAS-6_N_ (Leu-76 *versus* Ile-95 and Leu-137 *versus* Ile-151; Fig. S8G), which may be responsible for the increased methylpiperidine mobility in CrSAS-6_N_ MD simulations compared with those of HsSAS-6_N_. At the same time, we note that CrSAS-6_N_–A12 simulations clearly underestimate the fluctuations in the binding pose of this compound, because no relevant complex formation was observed *in vitro*. (Fig. 4*B* and Fig. S6B). Similarly, A11 binding in MD simulations appears less dynamic than experimental evidence would suggest. Although A11 titrations show that ligand binding and dissociation occur in the fast (sub-μs) NMR timescale (*e.g.* Fig. S3A), we observed no instance of A11 clearly leaving the binding pocket in any of the 0.5 μs-long simulations (Fig. S9, A–C and Movies S1–S3).

## Discussion

We presented here work toward the identification of small molecules targeting the SAS-6 head domain and preventing its dimerization. SAS-6 is the main component of the cartwheel scaffold in centrioles and in this role serves to ensure correct assembly of this organelle. Because SAS-6 oligomerization, including via self-association interactions of the protein head domain, is essential for centriole formation, a high-affinity and high-specificity compound blocking head domain dimerization could have a wide range of possible uses, from cell biological tool to putative anti-cancer therapeutic. However, further steps are necessary to develop such a potent and specific compound starting from this work.

Previously, a bromoindole derivative termed PK9119 was shown to bind the dimerization site of *Leishmania major* SAS-6 head domains with millimolar-level affinity and potentially also that of *D. rerio* SAS-6, although with significantly smaller potency (18). PK9119 did not bind CeSAS-6_N_ in our NMR assays (Fig. S10). Rather, in combined *in silico* and *in vitro* assays we demonstrated that compound A11 associates equally well with the head domains of nematode, algae, and human SAS-6, as judged by perturbations of NMR spectra (Fig. 2*D* and Fig. S5, A and D) and persistence in MD simulations (Fig. S9, A–C), whereas compound A12 shows binding specificity for the human orthologue. Thus, we consider that A11 may provide a starting point for developing a compound that would promiscuously target SAS-6 head domains. Furthermore, the presence of two SAS-6-binding molecules in our library of just 24 highly soluble compounds suggests that the pharmacophore model used captures preferences common across SAS-6 head domains. Thus, our relatively small compound library may comprise a good initial set of molecules for identifying binders of SAS-6 orthologues in general.

Binding of A11 to CeSAS-6_N_ was notably weak, with millimolar-level affinity (Fig. S3), which is likely responsible for the lack of large-scale changes in the CeSAS-6_N_ structure and dynamics inferred from MD simulations and {^1^H}-^15^N NOE NMR experiments (Fig. S4 and Movie S1). However, we were unable to sufficiently resolve peaks in the NMR spectra for protein dynamics from many residues in the CeSAS-6_N_ α1-α2 loop, including residues potentially forming hydrogen bonds to A11; thus, we cannot exclude the possibility that localized changes in α1-α2 loop dynamics may occur upon A1 binding. Nevertheless, because A11 was sufficiently potent to prevent protein oligomerization in AUC and EM assays (Fig. 3), we considered how compound affinity may be improved. We noted that, first, both A11 and A12 include a sulfonyl group that participates in hydrogen bonding interactions with residues of the protein α1-α2 loop (Fig. 5). In A11, an aminopropanol moiety extends from the sulfonyl group and provides an additional hydrogen bond via interactions with the α1-α2 loop, whereas in A12 similar functionality is offered by a methoxy-benzylacetamide moiety. Sulfonyl groups were present in several ligands identified as putative CeSAS-6_N_ binders (Table S1), stemming from the selection for hydrogen bond acceptors in the pharmacophore mode. However, some of these ligands were poorly soluble (A1, C8, and C9), which limited screening of sulfonyl derivatives. Nevertheless, we believe that neither the sulfonyl group nor its extensions present in A11 and A12 are optimal for the protein-ligand interactions observed, because of high levels of ligand mobility permitted by bond rotations around these groups. Rather, we propose that replacing the sulfonyl with a semi-rigid ester group, extended with a short alkylamine or alcohol moiety, may prove to be more favorable by reducing the number of possible conformational states for the ligand.

Both A11 and A12 feature aromatic groups, but only that of A11 interacted with the hydrophobic cavity of SAS-6 head domains (Fig. 5, *A*–*D*), likely because of hydrophilic decorations of the A12 aromatic moiety. The relevance of aromatic groups in SAS-6 head domain dimerization is well understood, not least because most SAS-6 proteins use a phenylalanine amino acid at the tip of the β6-β7 loop to mediate the key hydrophobic interaction (17, 19, 20), whereas a tryptophan substitution at the same site enhances the interaction strength (16, 23). Thus, an aromatic group with minimal and hydrophobic decorations, similar to that of A11, is likely essential for deriving a promiscuous binder of SAS-6 head domains. However, potentially more exciting is the discovery that cycloalkane groups, such as the methylpiperidine of A12, may also be suitable for interacting with the SAS-6 head domain dimerization cavity and confer specificity in the interaction. As seen in MD simulations of HsSAS-6_N_ with A12 (Fig. 5*E*), the available hydrophobic interface in this orthologue is larger than originally thought on the basis of other SAS-6 structures, and this interface could be exploited by “growing” the methylpiperidine moiety, thereby gaining human SAS-6-specific enthalpic contributions to the interaction. Further, at the periphery of the dimerization interface, two residues, Lys-86 and His-136 in HsSAS-6_N_, are only partly exploited by our currently ligand designs and offer opportunities for hydrogen bonding. Lys-86, in particular, is little-conserved among SAS-6 orthologues (Fig. S8G), suggesting that it could be targeted for interactions that would increase ligand specificity to human SAS-6.

In conclusion, we show here that a pharmacophore model derived from the dimerization interface of SAS-6 head domains can successfully identify compounds binding to this interface with the potential to block SAS-6 oligomerization. Intriguingly, although the pharmacophore was designed on the basis of general SAS-6 preferences, we observed the emergence of interaction specificity toward human SAS-6 already in the context of a relatively small library of compounds, likely as a result of packing contributions at the dimerization cavity. We propose that these contributions can be further optimized toward a strong and specific binder of human SAS-6.

## Experimental procedures

### Protein production and purification

Constructs encoding fragments of *C. elegans* SAS-6 (UniProt ID: O62479) residues 1–168 (CeSAS-6_N_) or 1–215 (CeSAS-6_N-CC_) were cloned in pHis vector (17), which provides an N-terminal His_6_-tag and thrombin cleavage site for removal of the cloning tag. A construct encoding *C. reinhardtii* SAS-6 (UniProt ID: A9CQL4) residues 1–159 (CrSAS-6_N_) was cloned in pSTCm1 vector (17), which also provides N-terminal His_6_-tag and thrombin cleavage elements. A construct encoding human SAS-6 (UniProt ID: Q6UVJ0) residues 1–152 (HsSAS-6_N_) was cloned in pFLOAT2 vector (39), which provides an N-terminal His_6_-tag and a human rhinovirus 3C protease cleavage site. Site-specific mutations were introduced in these constructs by the QuikChange method (Agilent Technologies). The identity of constructs was verified by sequencing (Eurofins Scientific).

*Escherichia coli* strain BL21(DE3) cells bearing these genetic constructs were grown at 37 °C in lysogeny broth, or M9 minimal medium supplemented with ^15^N-enriched NH_4_Cl or ^15^N-enriched NH_4_Cl and ^13^C-enriched glucose, and appropriate antibiotics. Protein expression was induced at optical density at 600 nm of ≥0.6 by addition of 0.25 mm isopropyl β-d-1-thiogalactopyranoside and let to proceed overnight at 18 °C. The cells were harvested by centrifugation and resuspended in 150 mm NaCl, 20 mm Na_2_HPO_4_, pH 7.5, buffer supplemented with 1% v/v Triton X-100 and cOmplete mini EDTA-free protease inhibitor mixture (Roche). The cell suspension was lysed by sonication and clarified by centrifugation at 30,000 × *g* at 4 °C for 30 min.

Clarified supernatants were loaded in pre-packed HiTrap Talon columns (GE Lifesciences) equilibrated in lysis buffer. The columns were washed in lysis buffer supplemented with 5 mm imidazole, and bound proteins were eluted from columns by a gradient of lysis buffer supplanted with 250 mm imidazole. His_6_-tags were removed by addition of thrombin (Sigma-Aldrich) or homemade human rhinovirus 3C protease, and protein samples were further purified by size exclusion chromatography using pre-packed Sephadex 75 or Sephadex 200 HiLoad 16/600 columns (GE Healthcare Life Sciences) equilibrated in NMR or crystallization buffer (see below). Protein-containing fractions were concentrated using spin ultrafiltration and Amicon Ultra (Millipore) devices at 4 °C. Protein quality was assessed by SDS-PAGE chromatography, protein identity was confirmed by electrospray MS (Department of Biochemistry, University of Oxford, UK), and protein concentration was established by UV spectroscopy using a calculated extinction coefficient (40) and a NanoDrop spectrophotometer (Thermo Fisher Scientific).

### NMR

Sequence-specific NMR resonance assignments were performed as described previously (41). Briefly, NMR experiments were performed at 25 °C using Bruker Avance III spectrometers with cryogenic TCI probeheads and 11.7–17.6-T magnetic field strengths. We used samples of ^13^C/^15^N-enriched proteins at 0.5 mm concentration for resonance assignments and ^15^N-enriched proteins at 0.1 mm concentration for binding assays. Samples were in 150 mm NaCl, 20 mm Na_2_HPO_4,_ pH 7.5 buffer supplemented with 5% v/v D_2_O, 0.02% w/v NaN_3_, and 50 μm 4,4-dimethyl-4-silapentane-1-sulfonic acid for reference purposes. Samples for binding assays contained either 5% v/v DMSO or the indicated concentration of compound supplemented with DMSO to reach a 5% v/v final concentration. Assignment experiments were performed using 3D CBCA(CO)NH, CBCANH, and HNCA pulse sequences. NMR data were processed using NMRpipe (42) and analyzed using PIPP (43) and Sparky (44). Where necessary, chemical shift assignments were transferred between WT and mutant constructs by overlaying spectra. For the calculation of combined chemical shift perturbations upon addition of compounds we summed up the ^1^H and ^15^N contributions using the following empirically derived equation:

$$\Delta \delta =\sqrt{\frac{1}{2}(\delta_{H}^{2}+0.14\delta_{N}^{2})}$$

Chemical shift changes were considered notable if they exceeded at least twice the intrinsic experimental error (2σ_exp_) or twice the standard deviation (2σ) of chemical shift perturbation values of all residues in the experiment. The intrinsic experimental error was calculated from the digital resolution of spectra to be ∼0.01 ppm. {^1^H}-^15^N NOE ratios were recorded using 0.5 mm protein concentration samples and analyzed as described previously (41).

### Analytical ultracentrifugation

AUC sedimentation velocity experiments were performed at the Research Complex at Harwell (Harwell, UK) using a Beckman Coulter ProteomeLab XL-I ultracentrifuge and An-50Ti or An-60Ti rotors (Beckman Coulter) with sapphire window inserts. Experiments were conducted at 15 °C and 35,000 rpm with data recorded simultaneously at a 280 nm wavelength and via interference optics over 250 complete scans. Protein samples were at 50 mm concentration in 150 mm NaCl, 20 mm HEPES buffer, pH 7.5, with 2 mm Tris(2-carboxyethyl)phosphine and supplemented with 5% v/v DMSO or small molecule compounds at 2 mm final concentration as indicated. Data were analyzed using Sedfit (45).

### EM

Electron micrographs were recorded as described previously (16). Briefly, freshly purified protein samples at 1 mg/ml concentration were extensively dialyzed against a 150 mm NaCl, 20 mm HEPES, pH 7.5 buffer, supplemented with 5% v/v DMSO or 2 mm A11 as necessary, and applied to carbon-coated grids, which were glow discharged using a Leica EM ACE200 vacuum coater. The samples were incubated on grids for 2 min prior to blotting and staining with 2% w/v uranyl acetate for 30 s before being left to dry in the dark for 10 min. Electron micrographs were obtained using a Tecnai12 transmission electron microscope and a Gatan OneView CMOS camera.

### X-ray crystallography

Crystals of HsSAS-6_N_ F131E were produced by the hanging drop vapor diffusion method. A Mosquito robot (TTP Labtech) was used to set up 100-nl drops with a 1:1 volume ratio of protein in 50 mm NaCl, 20 mm Tris(hydroxymethyl)aminomethane (Tris)-Cl, pH 7.5 buffer at 98 mg/ml concentration and diverse mother liquors. Crystals with plate-like morphology developed in ∼3 days using 30% w/v PEG 6000 and 0.1 m HEPES, pH 7 as mother liquor and were cryoprotected by brief immersion in mother liquor supplemented with 20% v/v glycerol prior to cooling in liquid nitrogen. Diffraction data were recorded at the Diamond Light Source (Harwell, UK) beamline I03 to 1.46 Å maximum resolution. Data were processed with Xia2 (46), and the structure was solved by molecular replacement using Phaser (47) and a homology model of HsSAS-6_N_ based on the previous *D. rerio* SAS-6 head domain structure (PDB ID: 2Y3V; 19). Iterative structure refinement was performed using Phenix (48) and model building in Coot (49). Crystallographic data quality and refinement statistics are shown in Table S2.

### Pharmacophore preparation and virtual screening

Discovery Studio 3.5 software (50) was used for the generation of the pharmacophore model using the CeSAS-6_N_ dimer crystallographic structure (PDB ID: 3PYI; 17) as the starting point. Water molecules in the structure were removed prior to analysis. Interaction patterns between the two domains were determined with a minimum feature distance of 1.5 Å. The pharmacophore model obtained was manually edited to contain a total of 10 features. We screened 100 different pharmacophore feature combinations, including at least five of these features in all combinations. At minimum, pharmacophore features included two hydrophobic or aromatic features close to Leu-98 and Ile-159 and one hydrogen bond donor. Further, we added exclusion features representing the van der Waals surface of CeSAS-6_N_.

The ∼13 million commercially available compounds of the Clean Drug–like subset of the ZINC database (51) were filtered according to molecular mass (300 to 500 Da), hydrogen bond donors (≥1), and aromatic groups (≥1) using OpenBabel (52), yielding a screening subset of ∼6 million molecules. 250 conformations of each compound were generated, fitted to the 100 different pharmacophore models with Discovery Studio 3.5, and ranked according to their fitting score as described before (53).

### Molecular dynamics simulations

A monomer from the crystal structure of CeSAS-6_N_ Δ103–130 (PDB ID: 4G79; 16) was used for preparation of the CrSAS-6_N_ starting model, with A11 and A12 inserted in the cavity of the dimerization site according to the pharmacophore model. For the CrSAS-6_N_ (PDB ID: 3Q0Y; 17) and HsSAS-6_N_ starting models with A11 and A12, we used monomer copies of the proteins and the best docking poses obtained by AutoDock Vina (54), using a 25 × 25 × 25-Å grid box, a search space of 10 binding modes, and a search parameter of 5. MD simulations were performed using GROMACS version 2018.3 (55) and the AMBER99SB-ILDN force field (56). The protein-ligand complexes were inserted in a pre-equilibrated box containing water implemented using the TIP3P water model (56). Force field parameters for A11 and A12 were generated using the general AMBER force field and HF/6–31G*–derived Restrained Electrostatic Potential atomic charges (57). The reference system consisted of the protein, the ligand, ∼16,900 water molecules, and 47 Na and 47 Cl ions in an 80 × 80 × 80-Å simulation box, resulting in a total number of ∼53,200 atoms. Each system was energy-minimized and subsequently subjected to a 10-ns MD equilibration, with an isothermal-isobaric ensemble using isotropic pressure control (58) and positional restraints on protein and ligand coordinates. The resulting equilibrated systems were replicated four times and independent 500-ns MD trajectories were produced at a constant temperature of 300 K, using separate v-rescale thermostats (58) for the protein, ligand, and solvent molecules. A time-step of 2 fs was used, and all bonds were constrained using the LINCS algorithm (59). Lennard-Jones interactions were computed using a cutoff of 10 Å and electrostatic interactions were treated using particle mesh Ewald (60) with the same real-space cutoff.

### Notes

Small molecule compounds were purchased from commercial vendors as indicated in Table S1 and dissolved in 100% v/v DMSO to a 40 mm final concentration. Structure figures were prepared using PyMOL (61). Sequence alignments were performed using Clustal Omega (62).

## Data availability

The HsSAS-6_N_ F131E structure model and associated data have been deposited in the RCSB Protein Data Bank under accession number 6Z4A. NMR assignments were deposited in BioMagResBank under accession numbers 50300 (CrSAS-6_N_ F145E) and 50308 (HsSAS-6_N_ F131D).
