# Supplementary material for: Identification of compounds that bind the centriolar protein SAS-6 and inhibit its oligomerization
Source: J Biol Chem. 2021 Jan 13;295(52):17922–34. doi: 10.1074/jbc.RA120.014780 (PMC7939395; doi:10.1074/jbc.RA120.014780)
Supplement: Supplementary file 1 [file mmc1.zip › 161454_2_supp_587423_qf77th.pdf]

Supporting information

Figure S1

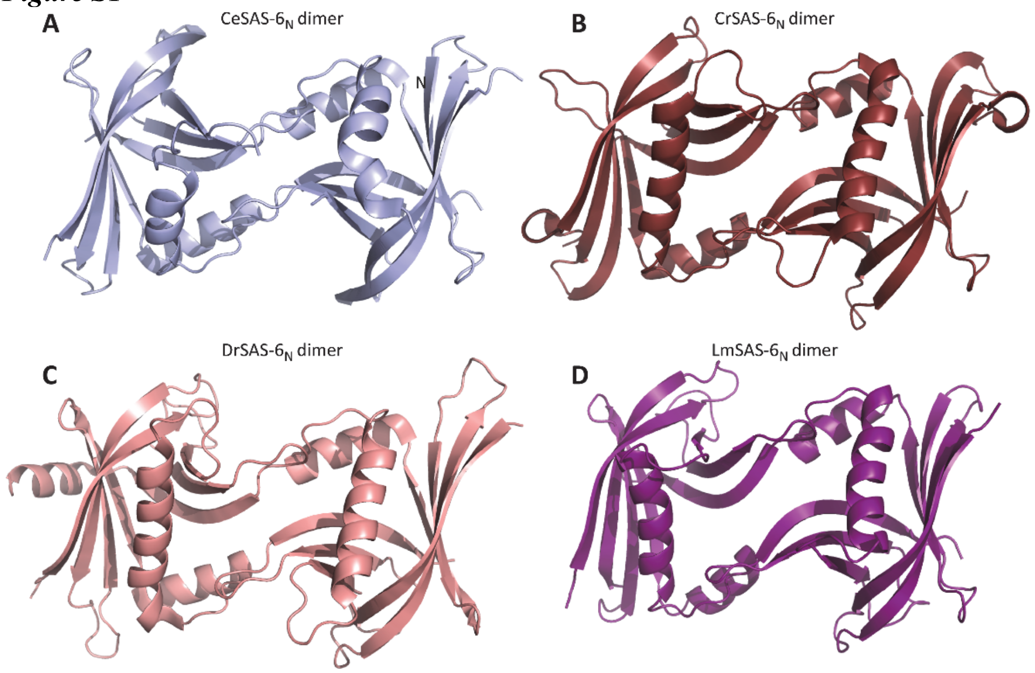

Supporting figure 1: Conserved dimerisation mode of SAS-6 head domains. Shown here are dimers of head domains from (A) *Caenorhabditis elegans* SAS-6 (CeSAS-6_N, derived from PDB ID 3PYI; 1), (B) *Chlamydomonas reinhardtii* SAS-6 (CrSAS-6_N, PDB ID 3Q0Y; 1), (C) *Danio rerio* SAS-6 (DrSAS-6_N, PDB ID 2Y3V; 2) and (D) *Leishmania major* SAS-6 (LmSAS-6_N, PDB ID 4CKM; 3). Dimerisation of head domains occurs in a conserved manner by inserting a single hydrophobic amino acid (I154 in CeSAS-6_N, F145 in CrSAS-6_N, F131 in DrSAS-6_N, F257 in LmSAS-6_N), into a hydrophobic cavity of the second monomer. The direct interaction interfaces of SAS-6 head domains, defined as all residues within 5 Å of the key hydrophobic amino acid, have an average pairwise C_α root-mean-square-deviation (RMSD) of 0.9 Å (0.4-1.6 Å range).

Figure S2

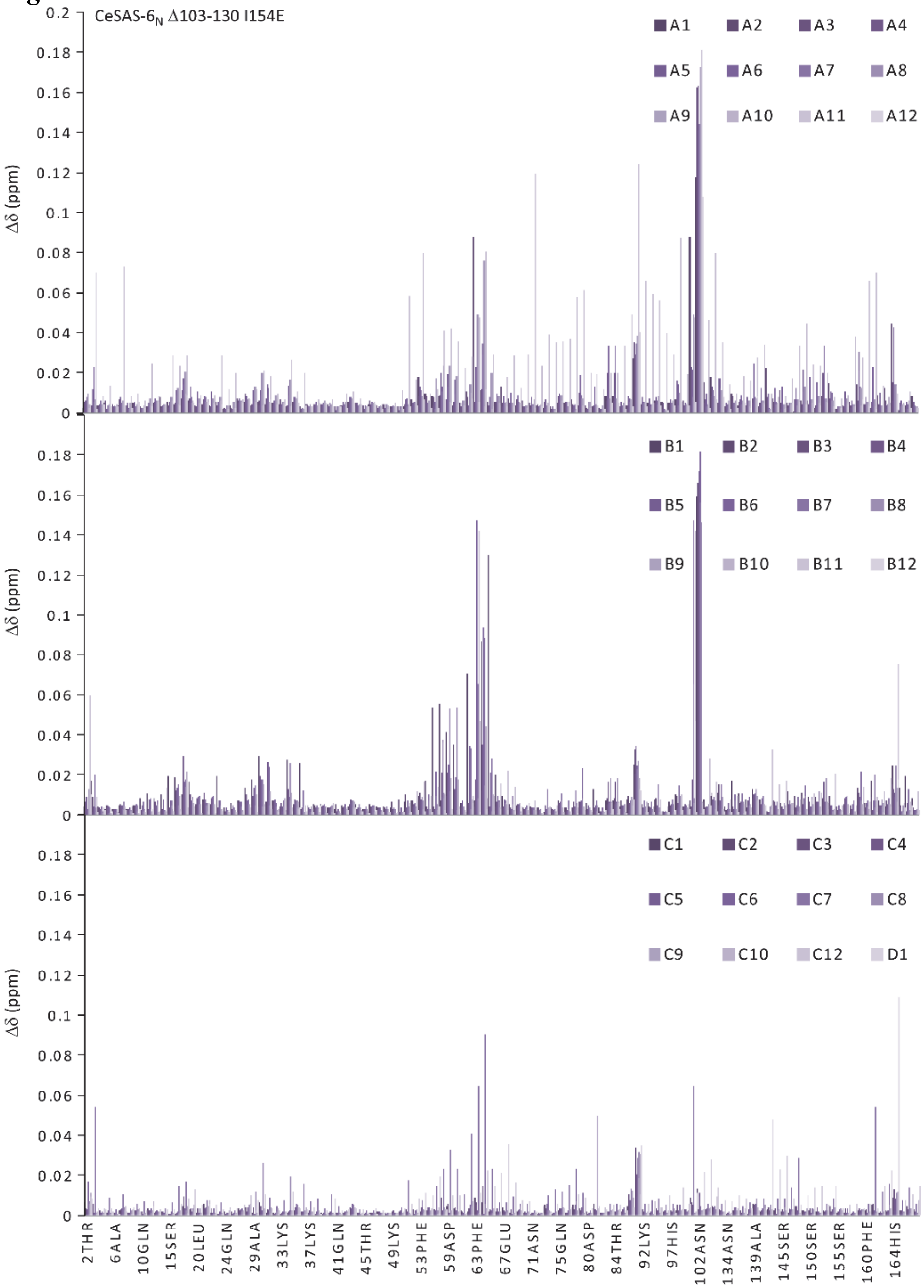

Supporting figure 2: Quantification of NMR assays for compound binding to CeSAS-6$_N$. Shown here are per-residue combined changes in ^{1}H and ^{15}N chemical shifts of CeSAS-6$_N$ Δ103-130 I154E resonances upon addition of 2 mM nominal compound concentration. We assessed 36 compounds suggested by the pharmacophore model to bind the hydrophobic cavity at the CeSAS-6$_N$ dimerisation interface.

Figure S3

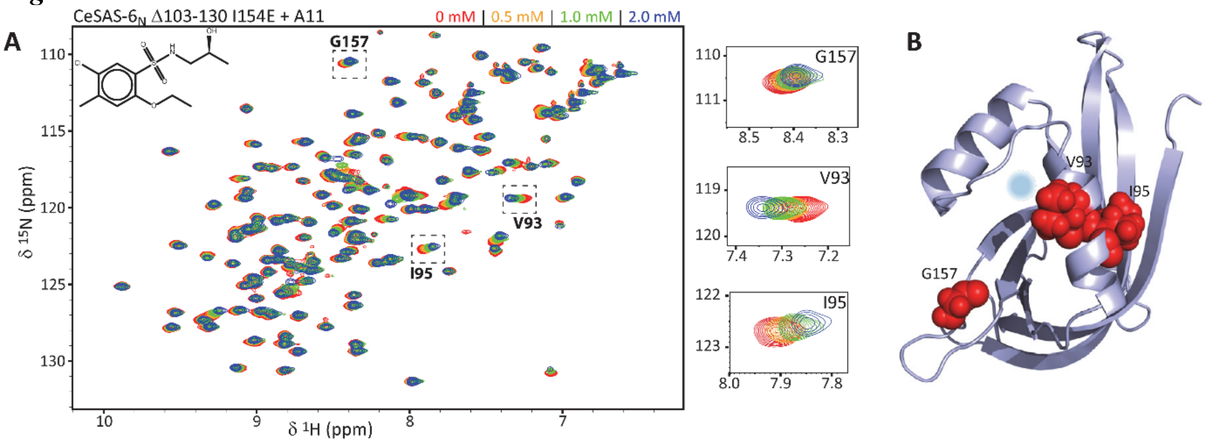

Supporting figure 3: NMR titration assays of A11 binding to CeSAS-6$_N$. A) Overlay of NMR ^{15}N HSQC spectra produced by CeSAS-6$_N$ Δ103-130 I154E alone (red) or in increasing concentrations of A11 (yellow: 0.5 mM, green: 1.0 mM, blue: 2.0 mM). The resonances of three amino acids at the hydrophobic cavity (V93 and I95) and the periphery (G157) of the dimerisation site are magnified. B) CeSAS-6$_N$ Δ103-130 monomer structure (PDB ID 4G79 (4)) showing the locations of the three highlighted amino acids relative to the dimerisation site (blue circle).

Figure S4

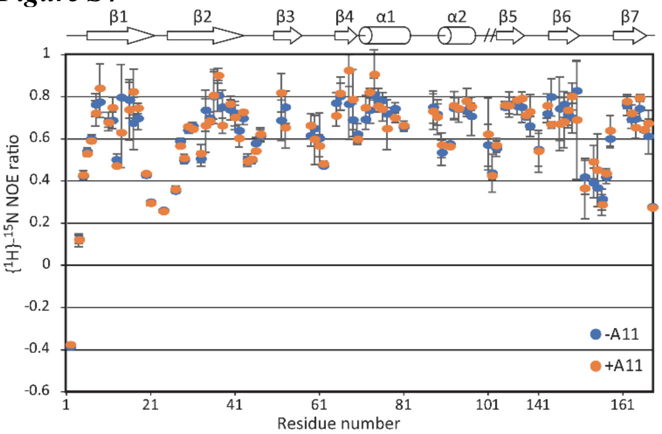

Supporting figure 4: Effect of ligand A11 on the fast-timescale dynamics of CeSAS-6$_N$. Shown here are per-residue plots of $\{^1H\}$-^{15}N heteronuclear NOE ratios of CeSAS-6$_N$ Δ103-130 I154E alone (blue) or in the presence of 2 mM A11 (orange). Error bars derive from the signal/noise ratios of spectra. The protein secondary structure is shown on top; a double forward stroke (//) denotes the position where the Δ103-130 excision takes place. Heteronuclear NOE ratios lower than ~0.6 indicate protein segments that are flexible in the picosecond-nanosecond timescale (5). Fast-timescale CeSAS-6$_N$ Δ103-130 I154E dynamics are not affected by addition of A11.

Figure S5

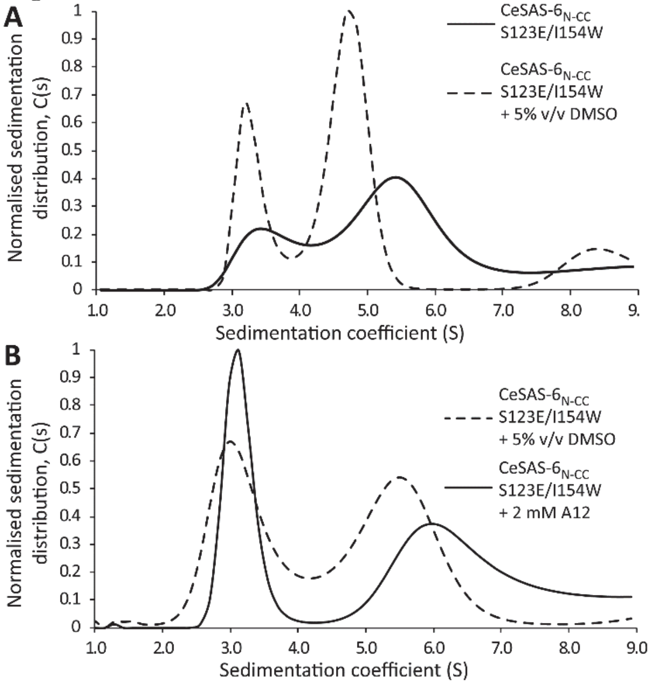

Supporting figure 5: Effect of DMSO and ligand A12 on CeSAS-6$_{N\text{-}CC}$ oligomerisation. Shown here are sedimentation velocity assays of CeSAS-6$_{N\text{-}CC}$ S123E/I154W in (A) aqueous buffer without (solid line) or with 5% v/v DMSO (dashed line) and (B) in aqueous buffer with 5% v/v DMSO (dashed line) or 5% v/v DMSO and 2 mM ligand A12 . Addition of DMSO decreases the presence of higher-order oligomeric species, suggesting that it interferes with CeSAS-6$_N$ dimerisation. Addition of A12 does not affect higher-order oligomerisation.

Figure S6

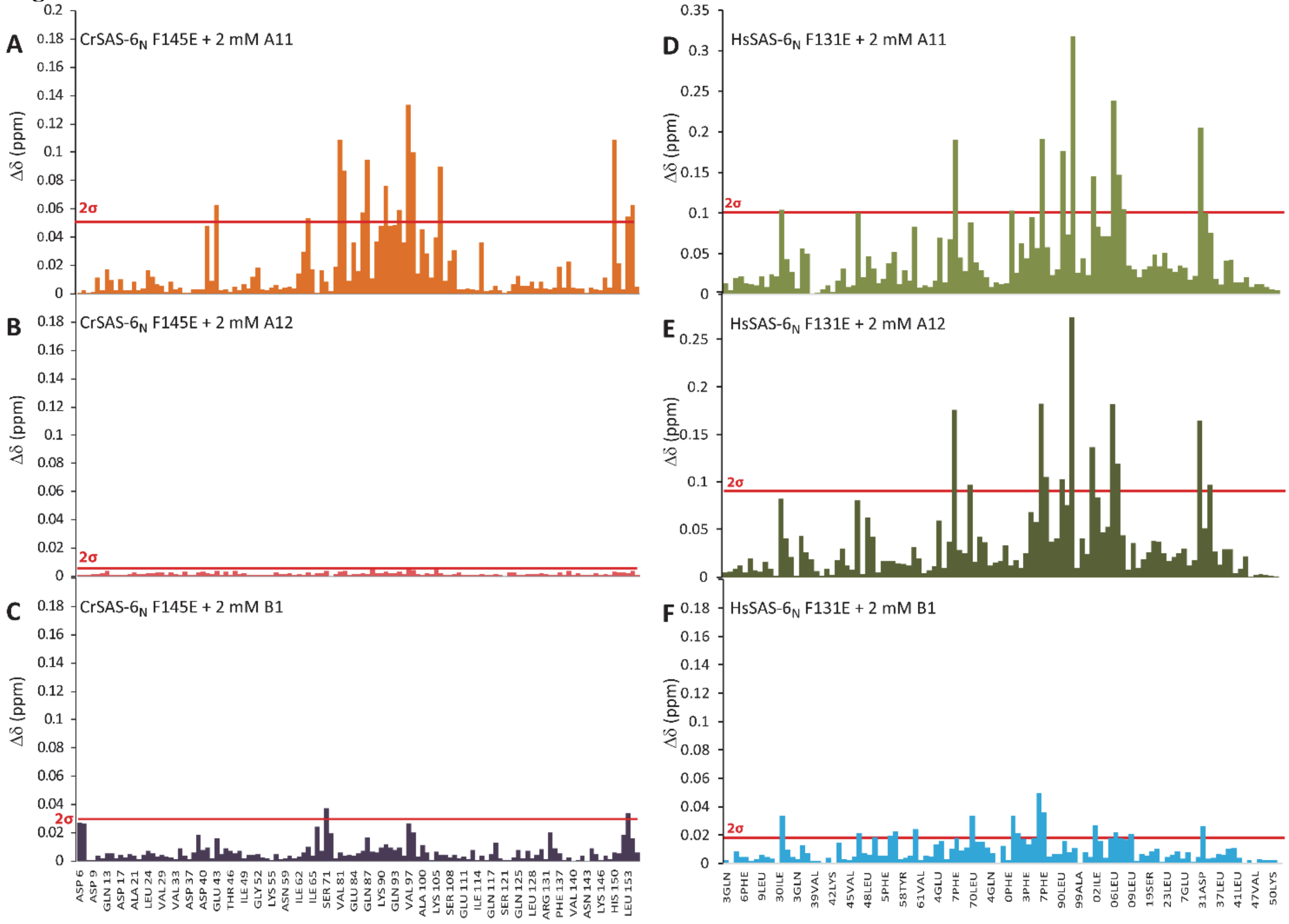

Supporting figure 6: A11, A12 and B1 binding to CrSAS-6$_N$ and HsSAS-6$_N$. Shown here are per residue combined changes in ^{1}H and ^{15}N chemical shifts of (A-C) CrSAS-6$_N$ F145E or (D-F) HsSAS-6$_N$ F131E resonances upon addition of 2 mM concentration of A11 (A,D), A12 (B,E) or B1 (C,F) compounds. A measure of two standard deviations of all changes observed is shown as red line, indicating amino acids that experienced the strongest perturbations upon compound addition.

Figure S7

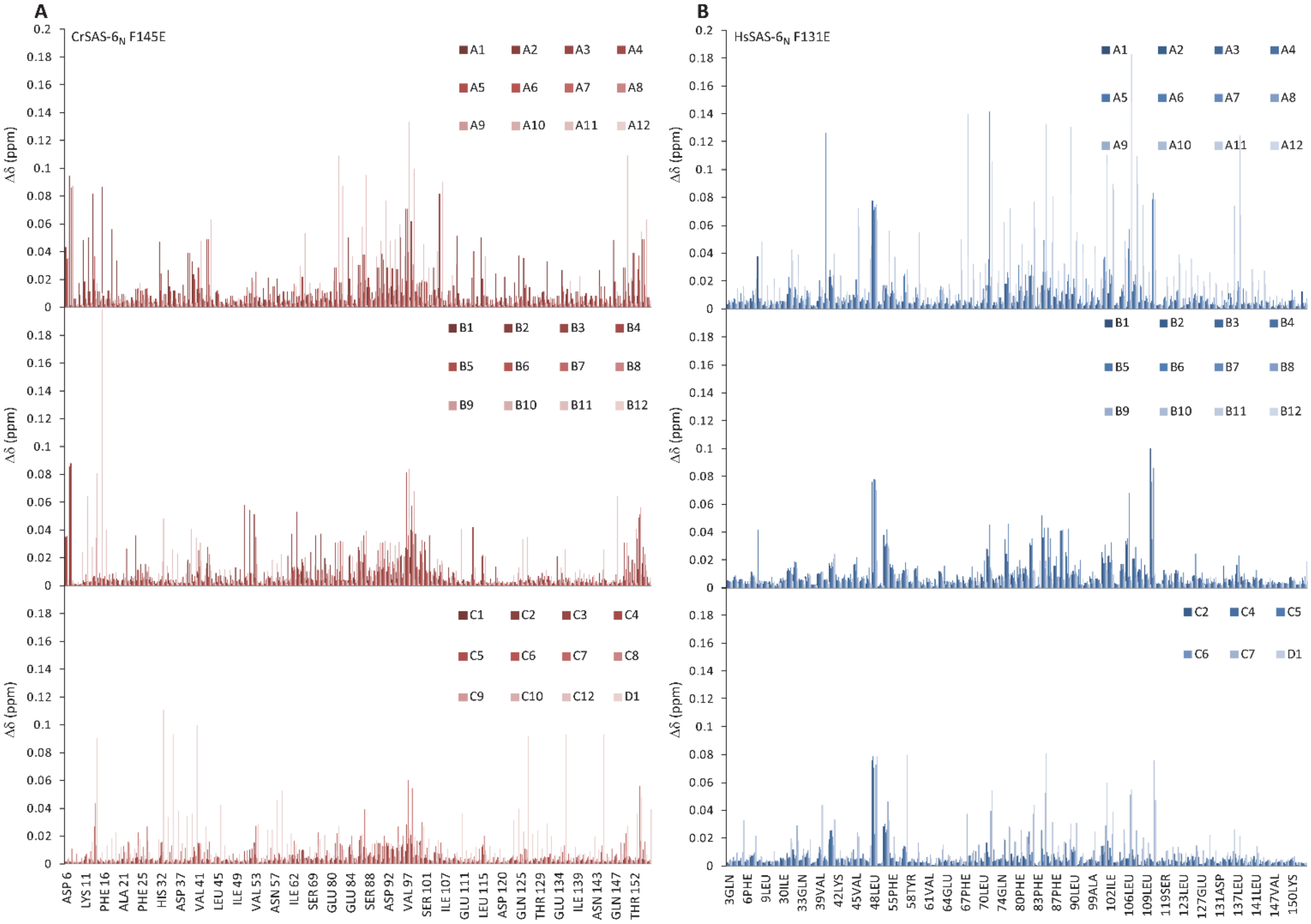

Supporting figure 7: Quantification of NMR assays for compound binding to CrSAS-6_N and HsSAS-6_N. Shown here are per residue combined changes in ^{1}H and ^{15}N chemical shifts of (A) CrSAS-6_N F145E or (B) HsSAS-6_N F131E resonances upon addition of 2 mM nominal compound concentration. For CrSAS-6_N F145E, we assessed 36 compounds originally suggested by the pharmacophore model as potentially binding the hydrophobic cavity of the CeSAS-6_N dimerisation interface. A subset of 30 compounds was tested against HsSAS-6_N F131E, excluding compounds that displayed no or very little solubility in aqueous buffers.

Figure S8

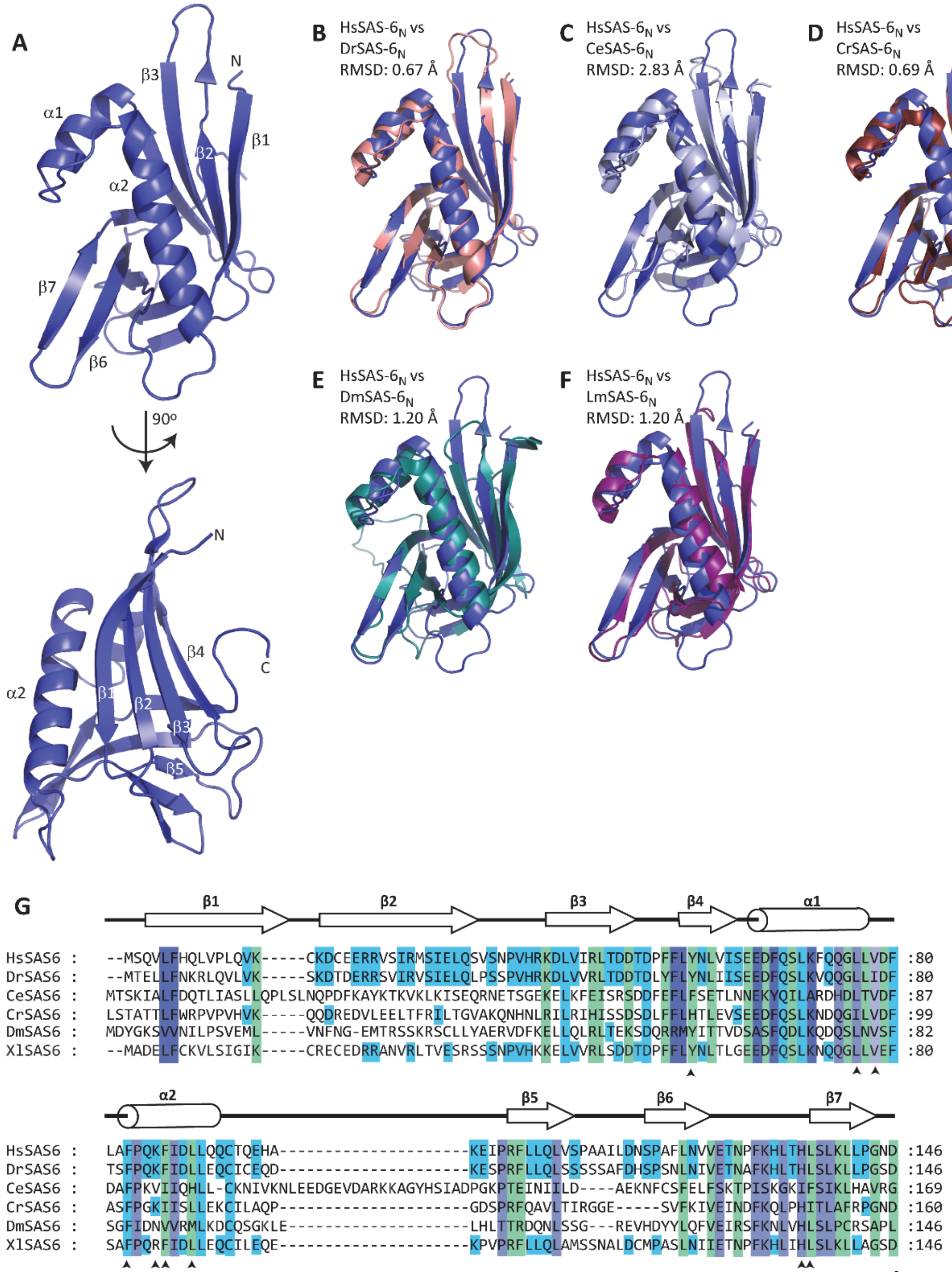

Supporting figure 8: Structure of the human SAS-6 head domain. A) The 1.46 Å crystallographic structure of HsSAS-6_N F131E monomeric variant is shown in two perpendicular views and schematic representation. The protein termini and secondary structure elements are indicated. Crystallographic data quality and refinement statistics are shown in Table S2. B-F) Superpositions of the HsSAS-6_N F131E domain structure (blue) with those of DrSAS-6_N (B, salmon, PDB ID 2Y3V; 2), CeSAS-6_N (C, light blue, 3PYI; 1), CrSAS-6_N (D, red, 3Q0Y; 1), *Drosophila melanogaster* SAS-6 head domain (E, teal, DmSAS-6_N, 5AL7; 6) and LmSAS-6_N (F, purple, 4CKM; 4). C_α RMSDs are shown. The overall fold of the SAS-6 head domain is conserved between humans and other species. Notable differences are the orientation of helices α1 and α2, and the length of α2, between HsSAS-6_N and CeSAS-6_N accounting for the increased structural divergence between these domains. G) Structure-assisted sequence alignment of SAS-6 head domains from diverse species. The secondary structure elements are shown on top. Residues conserved fully or in the majority of sequences are highlighted. Arrowheads below the sequences

denote amino acids of the head domain dimerisation cavity that form interactions with the A12 methylpiperidine group in molecular dynamics (MD) simulations. Sequences taken from UniProt IDs Q6UVJ0 (HsSAS-6), Q7ZVT3 (DrSAS-6), O62479 (CeSAS-6), A9CQL4 (CrSAS-6), Q9VAC8 (DmSAS-6) and Q6NRG6 (*Xenopus laevis* SAS-6, XlSAS-6).

Figure S9

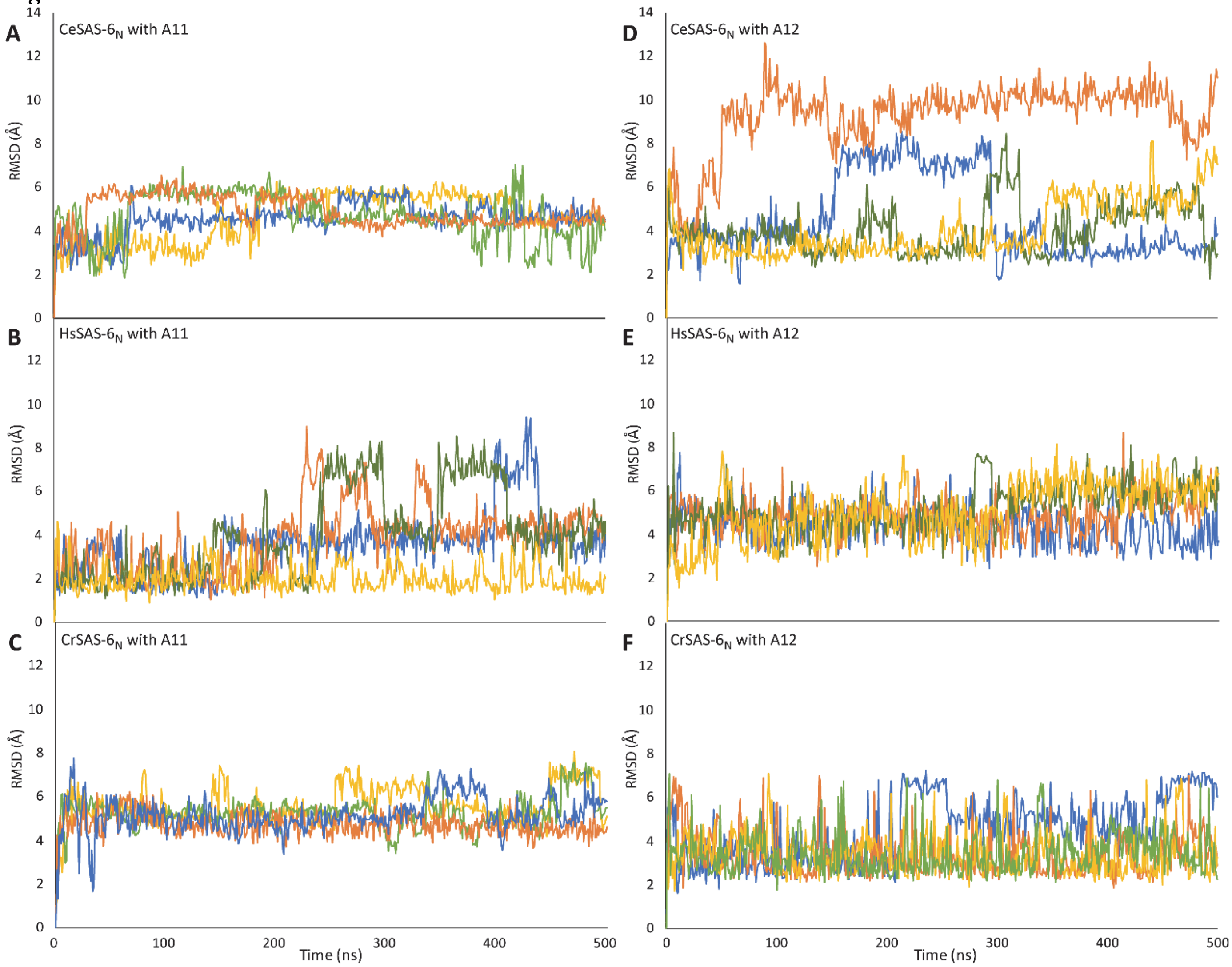

Supporting figure 9: MD simulations of A11 and A12 with SAS-6 head domains. A-C) Shown here are the all-atom ligand RMSDs from the start state observed during replicate 500 ns-long MD simulations of A11 with monomers of (A) CeSAS-6_N Δ103-130, (B) HsSAS-6_N or (C) CrSAS-6_N. RMSDs from the four simulation replicates are graphed in different colours. D-F) Similar graphs of all-atom ligand RMSDs from the start state for simulations of A12 with (D) CeSAS-6_N Δ103-130, (E) HsSAS-6_N or (F) CrSAS-6_N.

Figure S10

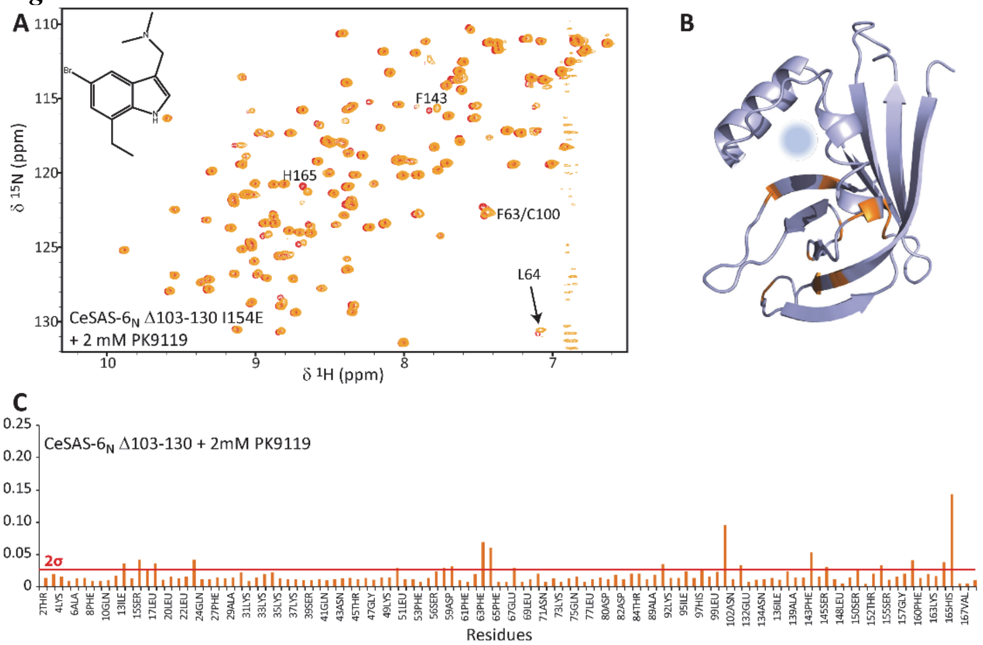

Supporting figure 10: NMR assay of CeSAS-6$_N$ with PK9119. A) Overlay of NMR ^{15}N HSQC spectra of CeSAS-6$_N$ Δ103-130 I154E alone (red) or in the presence of 2 mM PK9119 (yellow; 3). The structure of PK9119 is shown as insert. B) Per-residue quantification of combined changes in ^{1}H and ^{15}N chemical shifts of CeSAS-6$_N$ Δ103-130 I154E resonances upon addition of 2 mM PK9119. A measure of two standard deviations of all changes observed is shown as red line. C) CeSAS-6$_N$ Δ103-130 monomer structure with the dimerisation site targeted by compounds indicated by a light blue circle, derived from PDB ID 4G79 (4). The position of amino acids strongly perturbed by addition of PK9119 is shown in yellow.

Supporting movies S1-S6: MD simulations of A11 (Movies S1-S3) or A12 (Movies S4-S6) in complex with CeSAS-6_N (Movies S1 and S4), HsSAS-6_N (Movies S2 and S5) or CrSAS-6_N (Movies S3 and S6). SAS-6 domains are shown in schematic representations and ligands as sticks. Hydrogen bonds are indicated by dashed yellow lines.

Supporting tables

Table S1: List of candidate compounds for CeSAS-6_N binding

Name	Zinc ID	SMILES	Structure	2 mM aqueous solubility	Vendor
A1	ZINC71942314	CCCOc1cc(C)ccc1NC(=O)NCCN1CCOCC1	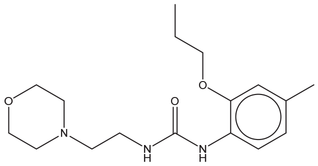	Soluble	ENAMINE Ltd.
A2	ZINC09970637	CCOC(=O)c1c(C)n(C)c2ccc(OC[C@@H](O)CNC)cc12	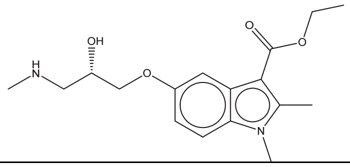	Soluble	Specs
A3	ZINC72414547	CCCOc1cc(C)c(C(C)C)cc1S(=O)(=O)NCC(C)C	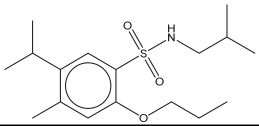	Insoluble	ENAMINE Ltd.
A4	ZINC70040105	CCCCn1c(SCCNS(C)(=O)=O)nc(C)c1C	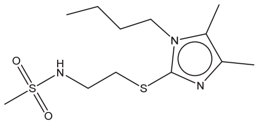	Soluble	ENAMINE Ltd.
A5	ZINC58247642	COC(=O)c1cc(NS(=O)(=O)c2cn[nH]c2)cc(C)c1C	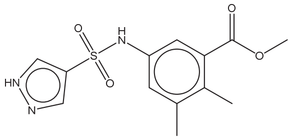	Soluble	ENAMINE Ltd.
A6	ZINC11487801	CCCCCn1c(SCC(=O)NC2CC2)nc2ccccc21	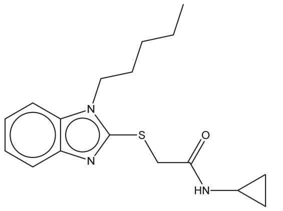	Soluble	ENAMINE Ltd.
A7	ZINC55414762	CC[C@@H](C)N(Cc1sccc1C)C(=O)c1cc(N)ccn1	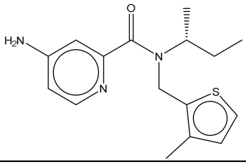	Soluble	ChemBridge Corporation
A8	ZINC54241093	COC(=O)c1c(NC(=O)C[C@@H]2NCCNC2=O)sc(C)c1C	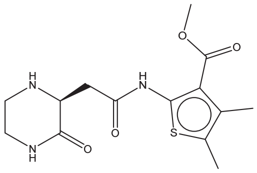	Soluble	Vitas-M Laboratory, Ltd.
A9	ZINC09584839	CC[C@H](C)Cc1c(C)sc2nc(CN3CCCC3)[nH]c(=O)c12	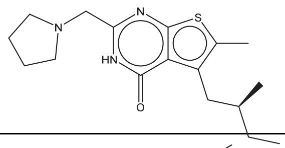	Insoluble	ENAMINE Ltd.
A10	ZINC13581301	CCN(CC)S(=O)(=O)c1cc(C(=O)NC)ccc1OC	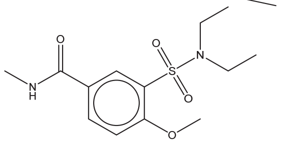	Soluble	Vitas-M Laboratory, Ltd.

A11	ZINC36638814	CCOc1cc(C)c(Cl)cc1S(=O)(=O)NC[C@H](C)O	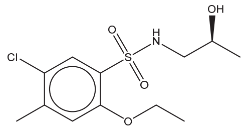	Soluble	Specs
A12	ZINC09737303	COc1ccc(NC(C)=O)cc1S(=O)(=O)N1CCCC[C@@H]1C	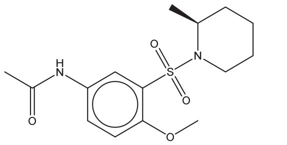	Soluble	UkrOrgSynthesis Ltd.
B1	ZINC06670228	CCOC(=O)c1c(NC(=O)NC[C@@H](C)O)sc2c1CCCC2	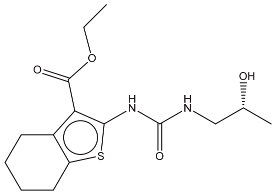	Soluble	Vitas-M Laboratory, Ltd.
B2	ZINC25772421	C[C@@H](NC(=O)[C@H](Cc1c[nH]c2ccccc12)NC(N)=O)C1CC1	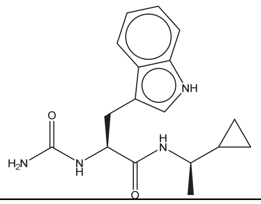	Soluble	ENAMINE Ltd.
B3	ZINC13477106	CCCCCn1c(SCCC)nc2c1c(=O)[nH]c(=O)n2C	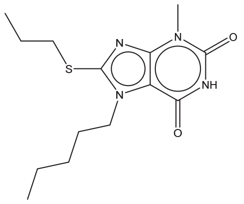	Insoluble	Vitas-M Laboratory, Ltd.
B4	ZINC12534617	Cc1ccccc1-n1c(C)nnc1SC[C@@H](O)c1ccccc1	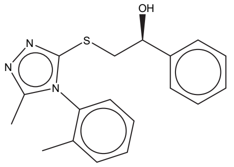	Soluble	ENAMINE Ltd.
B5	ZINC15693151	Cc1ccc(C)c([C@@H](C)NC(=O)CCNC(=O)c2ccco2)c1	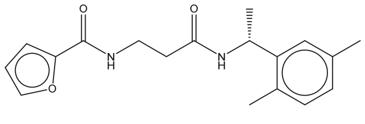	Soluble	ENAMINE Ltd.
B6	ZINC67921979	Cc1ccsc1-c1nc(Cn2cc(CCO)cn2)c(C)o1	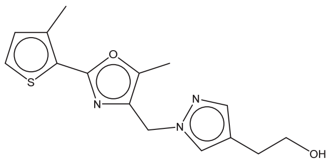	Soluble	ChemBridge Corporation
B7	ZINC81336504	CC(=O)NCc1ccc(C(=O)N2C[C@H](C)Oc3ccc(Cl)cc32)o1	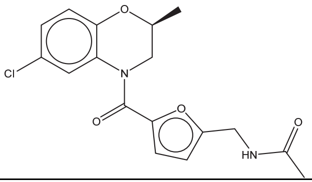	Soluble	UkrOrgSynthesis Ltd.
B8	ZINC65591069	CCOC(=O)c1cnn(CC(=O)N[C@H](C)c2cc(C)ccc2C)c1N	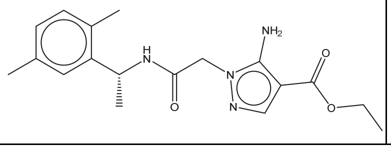	Soluble	ENAMINE Ltd.

B9	ZINC81048793	CN(Cc1cccc(C(N)=O)c1)C(=O)c1occc1Br	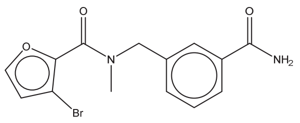	Soluble	UkrOrgSynthesis Ltd.
B10	ZINC69442707	CCN(CC)C(=O)c1cc(C2CC2)nc2c1c(=O)[nH]c(=O)n2CC	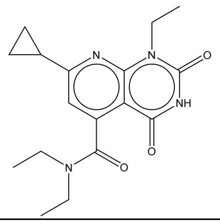	Soluble	ENAMINE Ltd.
B11	ZINC69870503	COc1cc(CN[C@H]2CCCC[C@@H]2O)cc(OC)c1OC(F)F	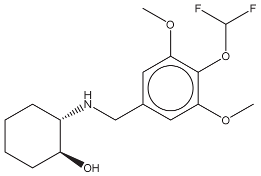	Soluble	ENAMINE Ltd.
B12	ZINC76202639	CC[C@@H](NC(=O)NC[C@@](C)(O)c1ccco1)c1c(C)nn(C)c1C	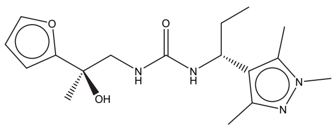	Soluble	UkrOrgSynthesis Ltd.
C1	ZINC28923015	Cc1nn(C)c(Cl)c1[C@@H]1Nc2ccccc2-c2nc3ccccc3n21	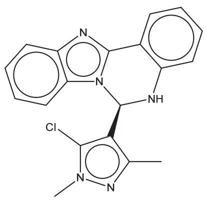	Insoluble	ENAMINE Ltd.
C2	ZINC22976783	CC(=O)N[C@H](CC(=O)N[C@H](C)c1ccccc1C)c1ccc(C)cc1	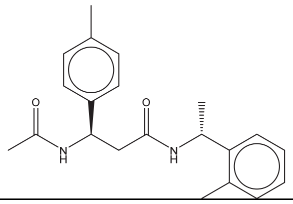	Partly soluble	UkrOrgSynthesis Ltd.
C3	ZINC00067653	CC[C@@H](C)Nc1nc2c(c(=O)[nH]c(=O)n2C)n1Cc1cccc(C)c1	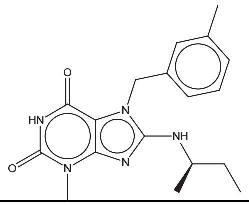	Insoluble	Vitas-M Laboratory, Ltd.
C4	ZINC79355337	Cn1cccc1[C@@H]1CCCN1C(=O)CNC(=O)NCc1ccco1	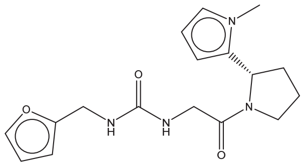	Soluble	UkrOrgSynthesis Ltd.
C5	ZINC04935336	CCOC(=O)[C@@H]1[C@@H](c2ccc(OC)c(OC)c2)c2c(O)n[nH]c2C[C@@]1(C)O	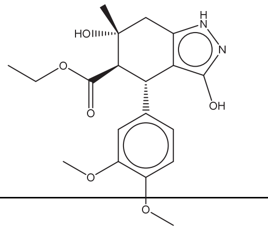	Soluble	Vitas-M Laboratory, Ltd.

C6	ZINC76041126	C=CCc1cc(C(=O)N[C@H](C)CCc2cccn2C)cc(OC)c1OC	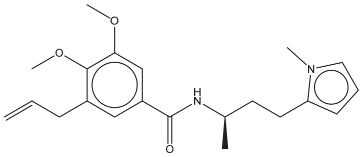	Partly soluble	ENAMINE Ltd.
C7	ZINC63846942	Cc1ccc2c(C(=O)N3CCC[C@H]3C)cc(-c3cnn(CCO)c3)nc2c1C	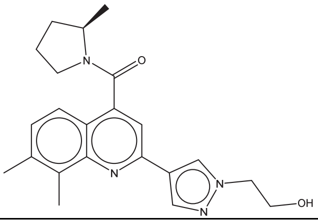	Soluble	ChemBridge Corporation
C8	ZINC64845906	Cc1noc(-c2c(C)sc(C)c2S(=O)(=O)NCc2ccccc2)c1C	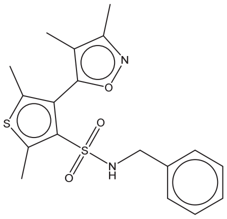	Partly soluble	ChemDiv, Inc.
C9	ZINC33306742	Cc1n[nH]c(-c2c(C)sc(C)c2S(=O)(=O)NC[C@H](C)c2ccccc2)c1C	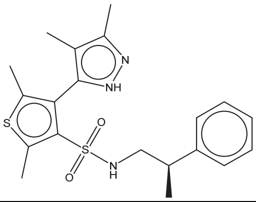	Insoluble	ChemDiv, Inc.
C10	ZINC02050114	CCCCC(=O)NC(Nc1sc2c(c1C(N)=O)CCCC2)(C(F)(F)F)C(F)(F)F	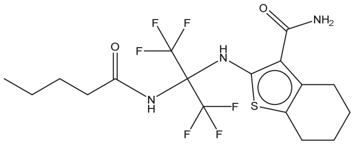	Partly soluble	Vitas-M Laboratory, Ltd.
C11	ZINC00678552	CC[C@H](C)Sc1nc2c(c(=O)[nH]c(=O)n2C)n1C[C@H](O)COc1ccc(Cl)cc1	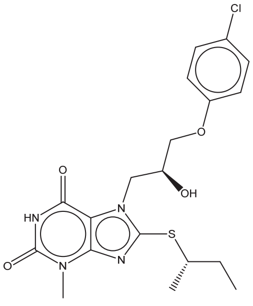	Insoluble	Specs
C12	ZINC13899084	CCN(CC)c1nc(NC2CCCCC2)nc(OC(C(F)(F)F)C(F)(F)F)n1	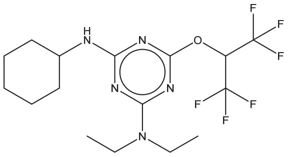	Insoluble	Vitas-M Laboratory, Ltd.

D1	ZINC12920785	O=C(O)Cc1csc(S[C@H](C(=O)N2CCCCC2)c2ccccc2)n1	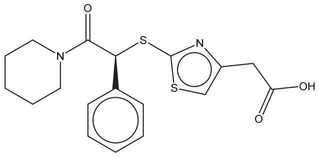	Partly soluble	ENAMINE Ltd.

Table S2: Crystallographic data collection and refinement statistics

Protein	*HsSAS-6$_N$ F131E*
PDB code	6Z4A
Space group	P 2_1 2_1 2
Unit cell (Å, °)	59.59 65.13 38.06<br>90.00 90.00 90.00
Beamline	DLS / I03
Wavelength (Å)	0.9795
Resolution range (Å)<br>High resolution shell (Å)	21.98 – 1.46<br>(1.49 – 1.46)
$R_{merge}{}^a$	0.060 (1.118)
$R_{meas}{}^a$	0.066 (1.238)
$R_{pim}{}^a$	0.027 (0.526)
Completeness[a] (%)	99.9 (99.5)
Multiplicity[a]	6.1 (5.4)
Mean I/σ(I)[a]	14.6 (1.4)
$CC_{½}{}^a$	0.999 (0.566)
Refinement statistics	
R_{work} (reflections)	19.9% (25,093)
R_{free}(reflections)	23.6% (1,247)
Number of atoms	
Protein atoms (excluding hydrogens)	1261
Hydrogens	1274
Ligands	6
Water	93
Average B factors (Å^2)	
Protein atoms (excluding hydrogens)	31.51
Hydrogens	31.24
Ligands	70.16
Water	41.03
RMSD from ideal values	
Bonds / angles (Å/°)	0.009 / 1.10
MolProbity statistics[b]	
Ramachandran favoured (%)	98.7%
Ramachandran disallowed (%)	0.0%
Rotamers favoured (%)	95.3%
Rotamers poor (%)	0.0%
Clashscore (percentile)	0.39 (100th)
MolProbity score (percentile)	0.64 (100th)

[a] Values in parentheses correspond to highest resolution shell.

[b] From MolProbity (7). Percentile scores compare the model to structures of comparable resolution; 100th percentile is the best among this set and 0th percentile is the worst.

Supporting references

1. Kitagawa, D., Vakonakis, I., Olieric, N., Hilbert, M., Keller, D., Olieric, V., Bortfeld, M., Erat, M. C., Flückiger, I., Gönczy, P., and Steinmetz, M. O. (2011) Structural basis of the 9-fold symmetry of centrioles. *Cell* **144**, 364-375
2. van Breugel, M., Hirono, M., Andreeva, A., Yanagisawa, H. A., Yamaguchi, S., Nakazawa, Y., Morgner, N., Petrovich, M., Ebong, I. O., Robinson, C. V., Johnson, C. M., Veprintsev, D., and Zuber, B. (2011) Structures of SAS-6 suggest its organization in centrioles. *Science* **331**, 1196-1199
3. van Breugel, M., Wilcken, R., McLaughlin, S. H., Rutherford, T. J., and Johnson, C. M. (2014) Structure of the SAS-6 cartwheel hub from *Leishmania major*. *Elife* **3**, e01812
4. Hilbert, M., Erat, M. C., Hachet, V., Guichard, P., Blank, I. D., Flückiger, I., Slater, L., Lowe, E. D., Hatzopoulos, G. N., Steinmetz, M. O., Gönczy, P., and Vakonakis, I. (2013) *Caenorhabditis elegans* centriolar protein SAS-6 forms a spiral that is consistent with imparting a ninefold symmetry. *Proc Natl Acad Sci U S A* **110**, 11373-11378
5. Kay, L. E., Torchia, D. A., and Bax, A. (1989) Backbone dynamics of proteins as studied by 15N inverse detected heteronuclear NMR spectroscopy: Application to staphylococcal nuclease. *Biochemistry* **28**, 8972-8979
6. Cottee, M. A., Muschalik, N., Johnson, S., Leveson, J., Raff, J. W., and Lea, S. M. (2015) The homo-oligomerisation of both Sas-6 and Ana2 is required for efficient centriole assembly in flies. *Elife* **4**, e07236
7. Chen, V. B., Arendall, W. B., 3rd, Headd, J. J., Keedy, D. A., Immormino, R. M., Kapral, G. J., Murray, L. W., Richardson, J. S., and Richardson, D. C. (2010) MolProbity: all-atom structure validation for macromolecular crystallography. *Acta Crystallogr D Biol Crystallogr* **66**, 12-21
